# Effects of potentilla discolor bunge extracts on oxidative stress and glycolipid metabolism in animal models of diabetes: a systematic review and meta-analysis

**DOI:** 10.3389/fphar.2023.1218757

**Published:** 2023-10-02

**Authors:** Yunjiao Yang, Wen Deng, Yue Wu, Changyan Zi, Qiu Chen

**Affiliations:** ^1^ Hospital of Chengdu University of Traditional Chinese Medicine, Chengdu, Sichuan, China; ^2^ School of Clinical Medicine, Chengdu University of Traditional Chinese Medicine, Chengdu, Sichuan, China; ^3^ Mianyang Attached Hospital of Chengdu University of Traditional Chinese Medicine, Mianyang, Sichuan, China

**Keywords:** potentilla discolor bunge, diabetes mellitus, flavonoids extracts, aqueous extracts, animal models, meta-analysis

## Abstract

**Background/aim:** Potentilla discolor Bunge (PDB) is an ancient herb of traditional Chinese medicine. Studies have suggested that extracts of PDB may ameliorate diabetes mellitus (DM). This study aimed to systematically assess the efficacy of PDB extracts on glycolipid metabolism and oxidative stress in animal models of diabetes and to provide evidence-based references for the use of PDB extracts.

**Methods:** This study followed the PRISMA 2020 guidelines. Studies were searched from eight databases until January 2023. Statistical analysis was performed using StataSE 15.0 and RevMan 5.3. The standard mean difference (SMD) and 95% confidence intervals (CI) were computed using the random-effects model. SYRCLE’s risk of bias tool was used to assess the risk of bias.

**Results:** In total, 32 studies with 574 animals were included. The findings demonstrated that PDB extracts considerably lowered fasting blood glucose (SMD: −3.56, 95%CI: −4.40 to −2.72, *p* < 0.00001); insulin resistance (SMD: −3.19, 95% CI: −5.46 to −0.92, *p* = 0.006), total cholesterol (SMD: −2.18, 95%CI: −2.89 to −1.46, *p* < 0.00001), triglyceride (SMD: −1.48, 95% CI: −2.01 to −0.96, *p* < 0.00001), low-density lipoprotein cholesterol (SMD: −1.80, 95% CI: −2.58 to −1.02], *p* < 0.00001), malondialdehyde (SMD: −3.46, 95% CI: −4.64 to −2.29, *p* < 0.00001) and free fatty acid levels (SMD: −3.25, 95%CI: −5.33 to −1.16, *p* = 0.002), meanwhile, increased insulin sensitivity index (SMD: 2.51 95% CI: 1.10 to 3.92, *p* = 0.0005), body weight (SMD:1.20, 95% CI: 0.38 to 2.01, *p* = 0.004), and the levels of high-density lipoprotein cholesterol (SMD: 1.04, 95% CI: 0.40 to 1.69, *p* = 0.001), superoxide dismutase (SMD:2.63, 95% CI: 1.53 to 3.73, *p* < 0.00001), glutathione peroxidase (SMD:1.13, 95%CI: 0.42 to1.83, *p* = 0.002), and catalase (SMD:0.75, 95% CI: 0.11 to 1.40], *p* = 0.02).

**Conclusion:** These findings suggest that PDB extracts can ameliorate DM by improving glycolipid metabolism and oxidative stress. PDB may be a promising medication for DM; however, due to significant heterogeneity between studies, these findings should be interpreted with caution. In addition, future well-designed trials should determine which components of the PDB play a major role in ameliorating DM and whether these benefits persist in humans.

**Systematic Review Registration:** https://www.crd.york.ac.uk/prospero, CRD42023379391

## 1 Introduction

Diabetes mellitus (DM) is characterized by abnormally increased blood glucose levels due to various etiologies. Approximately 700 million people will have DM by 2040, according to the International Diabetes Federation (Saeedi et al., 2019). DM is one of the most common chronic metabolic diseases. According to the latest data, metabolic diseases were responsible for 18.6 million deaths in 2019, with 43.6% increase since 1990. The burden of metabolic diseases has been on the rise for the past 30 years, especially among men and regions with low-middle socio-demographic index (Hu et al., 2023). Worldwide, nearly 90% of diabetic patients have type 2 diabetes mellitus (T2DM) (Zheng et al., 2018). Data from the global burden of metabolic diseases between 2000 and 2019 revealed that the prevalence rates of T2DM were 5,282 per 100,000 males, and 4,907 per 100,000 females, with an annual increase of 1.56% in global burden each year (1.64% in males and 1.51% in females) (Chew et al., 2023). Considering the high and rapidly increasing prevalence of DM, it imposes a heavy financial burden on healthcare systems. Except for hyperglycemia, DM causes complications, such as retinopathy, nephropathy, neuropathy, and atherosclerotic ischemia (Cundy et al., 2021; Slomski, 2022; Cai et al., 2023; Jonas et al., 2023). Additionally, patients with DM are more likely to suffer from neoplastic diseases, depression, and tuberculosis (Riza et al., 2014; Graham et al., 2020; He S. et al., 2022; Guo et al., 2022; Kramer et al., 2022; Ottaiano et al., 2022; Sharma et al., 2022). Despite the many effective medications developed, in contrast to expectations, the proportion of patients with well-controlled blood glucose has not increased (Nauck et al., 2021). This condition may be related to severe adverse drug reactions or other reasons (Taylor et al., 2015; Arcani et al., 2017; Vos and Rutten, 2017; Razavi-Nematollahi and Ismail-Beigi, 2019). Optimization of DM treatment remains a major public health issue worldwide. The search for new appropriate antidiabetic drugs remains an active area of research and development.

Currently, herbal medicines have been attracting attention worldwide and are increasingly used as complementary and alternative therapies for DM (Zhang and Jiang, 2012; Nie et al., 2019). *Fan Bai Cao* refers to Potentilla discolor Bunge (PDB), a member of the Rosaceae family distributed in the northern temperate zone. It was usually applied to treat hepatitis, diarrhea, or traumatic hemorrhage in the past (Tomczyk and Latté, 2009). However, the potential of PDB in treating metabolic diseases, especially DM, is becoming increasingly apparent (Zhang et al., 2010; Song et al., 2012; Li et al., 2014; Li et al., 2020; Luo et al., 2020). PDB contains more than ten types of constituents (Mou et al., 2020; Qin et al., 2020), among which eight can inhibit α-glucosidase (Gao et al., 2021). Seven triterpenoids have been isolated from PDB, and four can inhibit the protein tyrosine phosphatase-1B (PTP1B) that can prevent insulin receptor-insulin binding to cause insulin resistance and T2DM (Cui, 2016). Animal studies showed that water extract of PDB can improve glucose and lipid metabolism, enhance insulin sensitivity, promote glycogen synthesis, and inhibit gluconeogenesis (Li et al., 2020). The total flavonoids and triterpenoids from PDB had hypoglycemic and hypolipidemic effects and potent anti-oxidative stress properties in streptozotocin-and high-fat diet (HFD)-induced animal models of diabetes. (Zhang et al., 2010). Therefore, it is worthwhile investigating the effects of PDB extracts on DM.

Although existing studies suggest that PDB extracts have great potential in ameliorating DM, controversies existed among studies (Zhang et al., 2010; Li et al., 2014; Li et al., 2020). In addition, a systematic review and meta-analysis based on preclinical studies have not been conducted to synthesize evidence on the effects of PDB extracts on DM. Hence, this study aimed to conduct a comprehensive systematic review and meta-analysis to analyze the effects of PDB extracts on DM by pooling data from relevant animal studies. This study may provide evidence for future application of PDB extracts and determine future research direction.

## 2 Materials and methods

This study was registered on the PROSPERO platform (registration number: CRD42023379391) and was conducted according to the guidelines for preferred reporting items for systematic reviews and meta-analyses (Page et al., 2021). The checklist is available in Supplementary Material S1.

### 2.1 Search strategy

Two authors (YY and WD) independently searched PubMed, Wanfang database (Wanfang), Cochrane Library, Embase, Information Chinese Periodical Service Platform (VIP), Web of Science, China National Knowledge Internet (CNKI), and Baidu Academic database to identify relevant animal studies published in English and Chinese from inception till 30 January 2023, without publication time restriction. Additional eligible studies were identified by searching the references list of the included studies and in the unpublished gray literature. We used MeSH and free-text words appropriately adapted for each database. The following keywords were used (“diabetes mellitus”) AND (“ Potentilla discolor Bunge” OR “herba potentilla” OR “potentilla discolor decoction”). Supplementary Material S2 provides a detailed search strategy for PubMed.

### 2.2 Inclusion and exclusion criteria

#### 2.2.1 Inclusion criteria

1) participants: animals with diabetes regardless of their species, age gender, or disease induction method; 2) intervention: the experimental group was treated with PDB or its extracts regardless of timings, frequencies, and dosages; 3) control group: diabetic model animals induced by the same methods and treated with vehicle or no treatment; 4) outcomes: fasting blood glucose (FBG), total cholesterol (TC), insulin sensitivity index (ISI), body weight, fasting insulin (FINS), triglyceride (TG), high-density lipoprotein cholesterol (HDL-C), insulin resistance (IR), low-density lipoprotein cholesterol (LDL-C), malondialdehyde (MDA), nitric oxide synthase (NOS), superoxide dismutase (SOD), nitric oxide (NO), catalase (CAT), glutathione peroxidase (GSH-px), and free fatty acid (FFA).

#### 2.2.2 Exclusion criteria

1) the PDB extracts were mixed with other compounds of traditional Chinese medicine; 2) case reports; 3) therapeutic drugs were administered to the control group; 4) animals were not diabetic models; 5) conference paper; 6) lack of a control group; 7) review articles; and 8) *in vitro* or clinical studies.

### 2.3 Data extraction

The retrieved studies were managed using Endnote software (Version X9). Two authors (YY and WD) assessed the remaining studies after eliminating duplicates. Subsequently, titles and abstracts were screened, and the full-text version of potentially eligible studies was further reviewed. The following information was then used to create an Excel form: publication year, author, animal’s species, body weight and age, route of drug administration, intervention (form and dosage), sample size (intervention group/model group), methods for inducing DM, the modeling standard, the duration of treatment, and the outcomes. Disagreements were solved by consulting the corresponding author (QC). For studies with insufficient data, we emailed the authors to request specific information. We analyzed the data that were already available if there was no response.

### 2.4 Risk of bias assessment

Two researchers (YY and YW) independently assessed the bias of the included studies using the risk of bias tool of SYRCLE (Hooijmans et al., 2014). Sequence generation, baseline characteristics, assignment concealment, random housing, blinding of caregivers, investigators, and outcome assessors, random outcome assessment, incomplete outcome data, selective outcome reporting, and other types of bias were all included in the evaluation. Each item received a low, high, or uncertain ratings for its bias risk. If a study was judged to be low risk in one category, then the study received a point. Higher scores indicated higher quality. The corresponding author (QC) was consulted to discuss any disagreements.

### 2.5 Data synthesis and analysis

All analyses were performed using StataSE 15.0 and Review Manager 5.3. As we included various animal species and experimental models, a random effect model was used to pool the data. The SMD and 95% CI were used to illustrate the effect size. A *p*-value less than 0.05 was considered as the threshold for statistical significance. If the data were presented as a standard error, the standard deviation was calculated using the formula from the Cochrane Handbook for Systematic Reviews of Interventions (www.training.cochrane.org/handbook). I^2^ and Cochran’s Q statistics were used to assess the heterogeneity, and I^2^ > 50% and *p* < 0.05 indicated significant heterogeneity. To further explore the sources of heterogeneity, meta-regression was performed for the indicators containing 10 or more studies based on animal species, type of PDB extracts, treatment duration, and subgroup analyses were also conducted based on the types of PDB extracts, animal species, and treatment duration (2–4 weeks or 6–12 weeks). Sensitivity analyses were performed to verify the stability of the overall findings by progressively removing studies. Due to the limited number of studies reporting the IR, NO, NOS, and CAT levels, subgroup and sensitivity analyses were not conducted. Egger’s test was used to assess publication bias. The trim-and-fill method was used to assess the effect of publication bias on outcomes if the *p*-value in Egger’s test was less than 0.05.

## 3 Results

### 3.1 Search results

**FIGURE 1:**
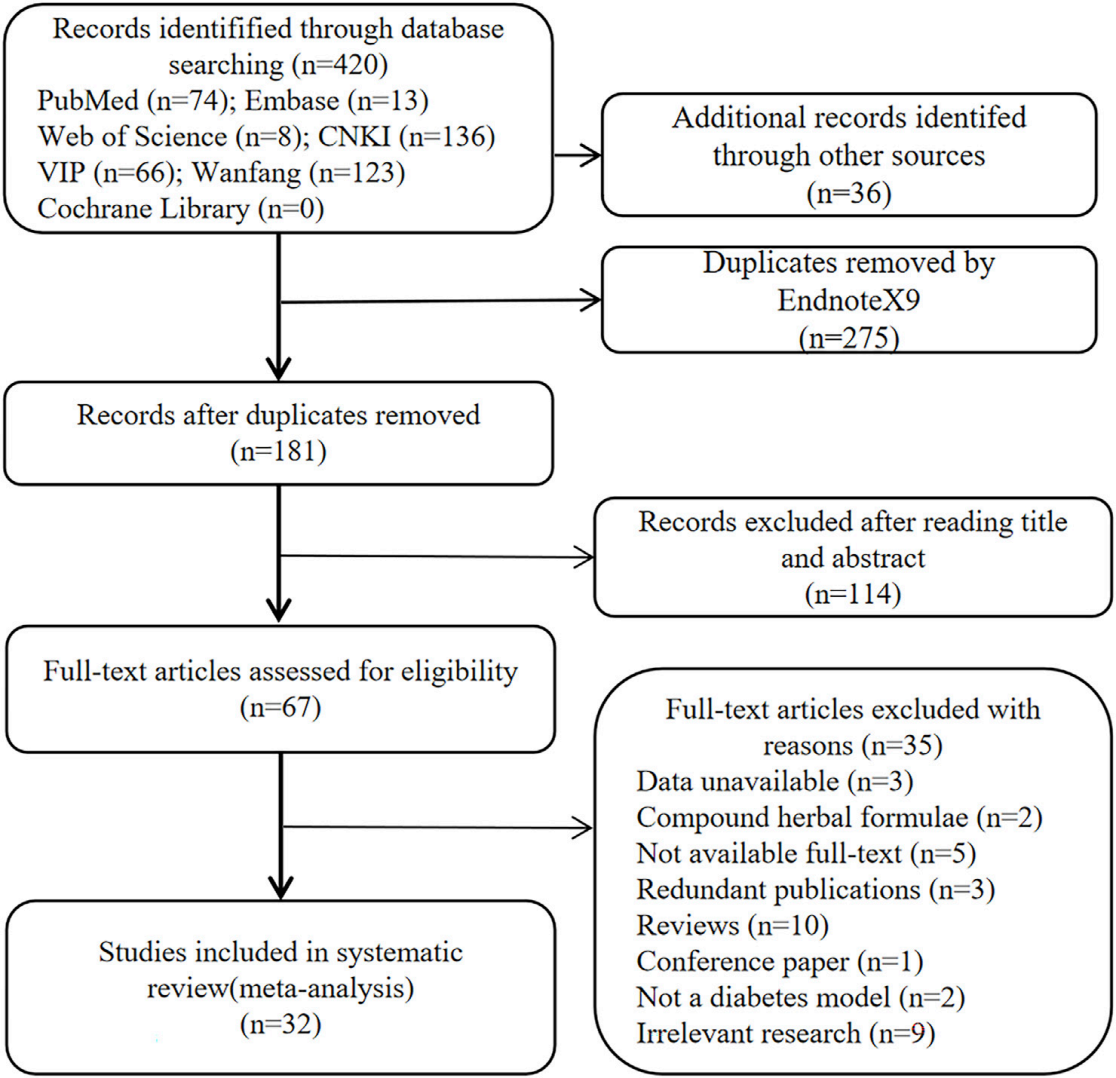
Flowchart for selection of studies.

In total, 456 studies were identified, including 74 from PubMed, 13 from Embase, eight from Web of Science, 0 from Cochrane Library, 136 from CNKI, 66 from VIP, 123 from Wanfang, and 136 from CNKI, and six from the Baidu Academic Database and references cited in the included studies. Using Endnote X9, 275 duplicates were removed. After reviewing the titles and abstracts, 114 articles were removed. Thirty-two studies with 574 animals were eventually included in this systematic review and meta-analysis after reviewing the full text of 67 papers. Figure 1 depicts the comprehensive selection procedure.

### 3.2 Characteristics of the included studies

The included studies were published between 2004 and 2021. The animal models included in these studies were rats or mouse models. Wistar rats were used in 16 studies (Guo and Cui, 2004; Li, 2004; Guo et al., 2005; Zhang and Shen, 2005; Bao et al., 2006; Cui et al., 2007; Hong et al., 2007; Piao et al., 2007; Zong et al., 2007; Ma and Cui, 2008; Sun et al., 2010; Yuan et al., 2010; Zhang et al., 2010; Cao and Zhang, 2011; Yan et al., 2012; Su et al., 2016); Sprague-Dawley rats (SD rats) were used in seven studies (Cheng and Li, 2011; Hu et al., 2014; Ding et al., 2016; Liu et al., 2016; Luo et al., 2020; Tan et al., 2020; Shi, 2021); C57BL/6 mice (db/db mice) were used in four studies (Yan et al., 2011; Li et al., 2014; Li et al., 2020; Kong et al., 2021). Two studies (Yan et al., 2011; Kong et al., 2021) used spontaneous T2DM model mice. Institute of Cancer Research (ICR) mice were used in one study (Li et al., 2017); obese-diabetic (Ob-db) mice were used in one study (Song et al., 2012); Kunming mice were used in three studies (Wang et al., 2008; Zeng et al., 2017; Qu et al., 2018). Animals’ weight ranged from 18 to 250 g. The gender of animals was not disclosed in two studies (Zhang and Shen, 2005; Zong et al., 2007). Nine studies used both male and female animals (Guo and Cui, 2004; Guo et al., 2005; Bao et al., 2006; Cui et al., 2007; Ma and Cui, 2008; Sun et al., 2010; Yuan et al., 2010; Cao and Zhang, 2011; Zeng et al., 2017), and the remaining studies used male animals. Fourteen studies (Guo and Cui, 2004; Guo et al., 2005; Bao et al., 2006; Cui et al., 2007; Ma and Cui, 2008; Yuan et al., 2010; Cao and Zhang, 2011; Yan et al., 2011; Yan et al., 2012; Li et al., 2014; Ding et al., 2016; Li et al., 2020; Luo et al., 2020; Kong et al., 2021) reported the age of the experimental animals, which ranged from 3 to 8 weeks. The length of treatment ranged from 2 to 8 weeks. Regarding the specific constituents of PDB composition, twenty studies (Guo and Cui, 2004; Li, 2004; Guo et al., 2005; Zhang and Shen, 2005; Bao et al., 2006; Cui et al., 2007; Hong et al., 2007; Piao et al., 2007; Zong et al., 2007; Ma and Cui, 2008; Yuan et al., 2010; Cao and Zhang, 2011; Yan et al., 2011; Song et al., 2012; Yan et al., 2012; Li et al., 2014; Ding et al., 2016; Zeng et al., 2017; Li et al., 2020; Luo et al., 2020) used aqueous extracts of PDB (the concentrated liquid extract of the whole plant after boiling) with doses ranging from 0.3 to 30 g/kg.d; Eleven studies (Wang et al., 2008; Sun et al., 2010; Zhang et al., 2010; Cheng and Li, 2011; Hu et al., 2014; Liu et al., 2016; Su et al., 2016; Li et al., 2017; Tan et al., 2020; Kong et al., 2021; Shi, 2021) used flavonoids from PDB with doses ranging from 0.054 to 36 g/kg.d; Two studies (Zhang et al., 2010; Qu et al., 2018) used triterpenes extracts from PDB with doses ranging from 0.501 to 0.3 g/kg.d. Concerning the diabetes induction model, most studies injected streptozotocin (15–100 mg/kg) in combination with a high-fat diet or a high-fat, high-sugar diet; One study (Li et al., 2014) used a high-fat diet alone to induce diabetes; Two studies (Hong et al., 2007; Piao et al., 2007) used two injections of alloxan to induce diabetes; Four studies (Li, 2004; Wang et al., 2008; Zeng et al., 2017; Qu et al., 2018) used a single injection of alloxan (120–200 mg/kg) to induce diabetes; One study (Song et al., 2012) used alloxan combined with high-fat diet. The majority of the included studies used FBG≥11.1 mmol/L as the threshold for successful modeling, and four studies (Cheng and Li, 2011; Ding et al., 2016; Luo et al., 2020; Shi, 2021) used FBG or random blood glucose (RBG) ≥ 16.7 mmol/L as the threshold for successful modeling. One study (Li et al., 2014) did not state the criteria for modeling. The details are presented in Table 1.

**TABLE 1:** Basic characteristics of the included studies.

Study	Animal	Drug route	Age (week)	Weight (g)	Gender	Number (I/M)	Intervention group	Model group	Duration	Induction method	Modeling standard	Outcomes	Intergroup differences
Yuan et al. (2010)	Wistar rat	Gavage	4	60	No restriction	7/7	Aqueous extract of PDB (30 g/kg·d)	Same volume of saline	4 weeks	HFSD + i.p.STZ (30 mg/kg)	11.1 mmol/L ≤ PBG	1.Body weight↑; 2.FBG↓; 3.FINS↑	1.*p* < 0.01; 2.*p* < 0.01 3.*p* < 0.01
Bao et al. (2006)	Wistar rat	Gavage	8	200 ∼ 250	No restriction	10/10	Aqueous extract of PDB (6 g/kg.d)	Same volume of saline	8 weeks	HFSD + i.v.STZ (15 mg/kg)	11.1 mmol/L < FBG <33.3 mmol/L	1.NO↓; 2.MDA↓; 3.NOS↓; 4.SOD↑	1.*p* < 0.05; 2.*p* < 0.05 3.*p* > 0.05; 4.*p* < 0.05
Piao et al. (2007)	Wistar rat	Gavage	NR	190 ∼ 230	Male	10/10	Aqueous extract of PDB (9 g/kg.d)	Same volume of distilled water	4 weeks	i.p.Alloxan (120 mg/kg) +(100 mg/kg). Two injections in total	11.1 mmol/L < FBG	1.FBG↓; 2.TC↓; 3.TG↓	1.*p* < 0.05; 2.*p* < 0.05 3.*p* < 0.05
Zhang et al. (2010)	Wistar rat	Gavage	NR	200–250	Male	10/10	Flavonoids from PDB (369 mg/kg.d) Triterpenes extract from PDB (501 mg/kg.d)	0.5% sodium carboxymethylcellulose	15 days	HFD + i.v.STZ (35 mg/kg)	7.0 mmol/L < FBG < 33.3 mmol/L	1.TC↓; 2.TG↓; 3.LDL-C↓; 4.HDL-C↑; 5.NO↓; 6.MDA↓; 7.SOD↑; 8.GSH-px↑; 9.Body weight↓	1.*p* < 0.01; 2.*p* < 0.01 3.*p* < 0.01; 4.*p* < 0.01 5.*p* < 0.01; 6.*p* < 0.01 7.*p* < 0.01; 8.*p* < 0.01; 9.*p* < 0.01
Cao et al. (2011)	Wistar rat	Gavage	8	NR	No restriction	10/10	Aqueous extract of PDB (18 g/kg.d)	Same volume of saline	8 weeks	HFD + i.v.STZ (18 mg/kg)	11.1 mmol/L < FBG	1.FBG↓; 2.FINS↓; 3.TC↓; 4.TG↓; 5.ISI↑	1.*p* < 0.05; 2.*p* < 0.01 3.*p* < 0.05; 4.*p* < 0.05 5.*p* < 0.05
Cui et al. (2007)	Wistar rat	Gavage	8	200 ∼ 250	No restriction	10/10	Aqueous extract of PDB (12 g/kg.d)	Same volume of saline	8 weeks	HFSD + i.v.STZ (15 mg/kg)	11.1 mmol/L < FBG<33.3 mmol/L	ISI↑	*p* < 0.01
Guo et al. (2004)	Wistar rat	Gavage	8	200 ∼ 250	No restriction	10/10	Aqueous extract of PDB (12 g/kg.d)	Same volume of saline	8 weeks	HFSD + i.v.STZ (15 mg/kg)	11.1 mmol/L ≤ FBG<33.3 mmol/L	FINS↓	*p* < 0.01
Guo et al. (2005)	Wistar rat	Gavage	8	200 ∼ 250	No restriction	10/10	Aqueous extract of PDB (12 g/kg.d)	Same volume of saline	8 weeks	HFSD + i.v.STZ (30 mg/kg)	11.1 mmol/L ≤ FBG < 33.3 mmol/L	1.TG↓; 2.TC↓; 3.HDL-C↓; 4.LDL-C↓	1.*p* < 0.01; 2.*p* < 0.01 3.*p* < 0.05; 4.*p* < 0.05
Ma et al. (2008)	Wistar rat	Gavage	8	200 ∼ 250	No restriction	10/10	Aqueous extract of PDB (90 g/kg.d)	Same volume of saline	8 weeks	HFSD + i.v.STZ (15 mg/kg)	11.1 mmol/L < FBG < 33.3 mmol/L	1.MDA↓; 2.GSH-px↑; 3.SOD↑; 4.CAT↑	1.*p* < 0.01; 2.*p* < 0.01 3.*p* < 0.01; 4.*p* < 0.05
Zong et al. (2007)	Wistar rat	Gavage	NR	180 ∼ 220	NR	10/10	Aqueous extract of PDB (12 g/kg.d)	Same volume of water	8 weeks	HFD + i.v.STZ (20 mg/kg)	11.1 mmol/L ≤ FBG<30 mmol/L	FINS↓	*p* < 0.01
Wang et al. (2008)	Kunming mice	Gavage	NR	18 ∼ 22	Male	10/8	Flavonoids from PDB (216 mg/kg.d)	Each mouse was given 0.4 mL normal saline	2 weeks	i.p.Alloxan (120 mg/kg)	11.1 mmol/L ≤ FBG	1.FBG↓; 2.FINS↑; 3.Body weight↑	1.*p* < 0.05; 2.*p* < 0.05 3.*p* < 0.05
Hong et al. (2007)	Wistar rat	Gavage	NR	190 ∼ 230	Male	10/10	Aqueous extract of PDB (9 g/kg.d)	Same volume of distilled water	4 weeks	i.p.Alloxan (120 mg/kg) +(100 mg/kg). Two injections in total	11.1 mmol/L ≤ FBG	1.FBG↓; 2.TC↓; 3.TG↓	1.*p* < 0.05; 2.*p* < 0.05 3.*p* < 0.05
Hu et al. (2014)	SD rat	Gavage	NR	180 ∼ 220	Male	8/8	Flavonoids from PDB (216 mg/kg.d)	Same volume of water	4 weeks	HFSD + i.v.STZ (30 mg/kg)	11.1 mmol/L ≤ FBG	1.Body weight↑; 2.FBG↓; 3.TG↓; 4.TC↓; 5.HDL-C↑; 6.LDL-C↓; 7.FINS↓; 8.IR↓; 9.FFA↓	1.*p* < 0.05; 2.*p* < 0.05 3.*p* > 0.05; 4.*p* < 0.05 5.*p* < 0.05; 6.*p* > 0.05 7.*p* < 0.05; 8.*p* < 0.05 9.*p* > 0.05
Ding et al. (2016)	SD rat	Gavage	4	120-140	Male	7/7	Aqueous extract of PDB (4 g/kg.d)	Same volume of water	4 weeks	HFD + i.p.STZ (35 mg/kg)	16.7 mmol/L ≤ RBG	1.Body weight↑; 2.RBG↓; 3.TG↓; 4.TC↓; 5.HDL-C↓; 6.LDL-C↓	1.*p* > 0.05; 2.*p* < 0.01 3.*p* > 0.05; 4.*p* < 0.05 5.*p* > 0.05; 6.*p* > 0.05
Cheng et al. (2011)	SD rat	Gavage	NR	160 ∼ 200	Male	10/10	Flavonoids from PDB (320 mg/kg.d)	Same volume of distilled water	12 weeks	HFSD + i.p.STZ (40 mg/kg)	16.7 mmol/L ≤ RBG	1.FBG↓; 2.FINS↓; 3.ISI↑; 4.CAT↑; 5.MDA↓; 6.GSH-px↑	1.*p* < 0.01; 2.*p* < 0.05 3.*p* < 0.05; 4.*p* < 0.05 5.*p* < 0.01; 6.*p* < 0.01
Yan et al. (2011)	C57BL/KsJ-db/db Mice	Gavage	6	40.4 ∼ 49	Male	6/6	Aqueous extract of PDB (400 mg/kg.d)	Same volume of sterile water	4 weeks	Spontaneous type 2 diabetic mellitus	-	1.FBG↓; 2.FINS↓; 3.ISI↑	1.*p* < 0.05; 2.*p* < 0.05 3.*p* < 0.05
Yan et al. (2012)	Wistar rat	Gavage	3	49 ∼ 57	Male	7/7	Aqueous extract of PDB (400 mg/kg.d)	Sterile water (10 mL/kg.d)	4 weeks	HFD + i.p.STZ (35 mg/kg)	11.1 mmol/L < FBG<33.3 mmol/L	1.Body weight↑; 2.FBG↓; 3.TG↓; 4.TC↓; 5.HDL-C↑; 6.LDL-C↓; 7. FFA↓	1.*p* < 0.05; 2.*p* < 0.05 3.*p* < 0.05; 4.*p* < 0.05 5.*p* < 0.05; 6.*p* < 0.05 7.*p* < 0.05
Liu et al. (2016)	SD rat	Gavage	NR	180 ∼ 220	Male	10/10	Flavonoids from PDB (54 mg/kg.d)	Giving 2 mL drinking water	4 weeks	HFSD + i.p.STZ (30 mg/kg)	11.1 mmol/L < FBG	1.FBG↓; 2.FINS↓; 3.MDA↓; 4.SOD↑; 5.GSH-px↑	1.*p* < 0.05; 2.*p* < 0.05 3.*p* < 0.05; 4.*p* < 0.05 5.*p* < 0.05
Zhang et al. (2005)	Wistar rat	Gavage	NR	NR	NR	10/10	Aqueous extract of PDB (12 g/kg.d)	Same volume of saline	8 weeks	HFSD + i.v.STZ (15 mg/kg)	11.1 mmol/L ≤ RBG<33.3 mmol/L	1.NOS↑; 2.NO↑	1.*p* < 0.01; 2.*p* < 0.01
Li et al. (2014)	C57BL/6 mice	Free feeding	4	NR	Male	6/6	HFD + Aqueous extract of PDB (The dose of PDB was not disclosed)	HFD alone	8 weeks	HFD	NR	1.Body weight↓; 2.FBG↓; 3.FINS↓; 4.IR↓; 5.TG↓; 6.TC↓; 7.LDL-C↓; 8.HDL-C↑	1.*p* < 0.05; 2.*p* < 0.05 3.*p* < 0.05; 4.*p* < 0.05 5.*p* < 0.05; 6.*p* < 0.05 7.*p* < 0.05; 8.*p* < 0.05
Li et al. (2017)	ICR mice	Gavage	NR	28–32	Male	8/8	Flavonoids from PDB (100 mg/kg.d)	Giving saline (0.1 mL/10 g)	6 weeks	HFD + i.p.STZ (100 mg/kg)	11.1 mmol/L < FBG	FBG↓	*p* < 0.001
Li et al. (2020)	C57BL/6J mice	Gavage	5	NR	male	8/8	Aqueous extract of PDB (400 mg/kg.d)	Same volume of saline	8 weeks	HFD + i.p.STZ (40 mg/kg)	11.1 mmol/L < FBG	1.Body weight↑; 2.FINS↑; 3.TG↓; 4.TC↓; 5.HDL-C↓; 6.LDL-C↓; 7.FFA↓	1.*p* < 0.05; 2.*p* < 0.05 3.*p* < 0.05; 4.*p* < 0.05 5.*p* < 0.05; 6.*p* < 0.05 7.*p* < 0.05
Li et al. (2004)	Wistar rat	Gavage	NR	190 ∼ 210	male	10/10	Aqueous extract of PDB (5.4 g/kg.d)	Same volume of distilled water	3 weeks	i.p.Alloxan (120 mg/kg)	10.mmol/L < FBG	1.FBG↓; 2.MDA↓; 3.SOD↑	1.*p* < 0.01; 2.*p* < 0.05 3.*p* < 0.01
Zeng et al. (2017)	Kunming mice	Gavage	NR	25 ∼ 30	No restriction	10/10	Aqueous extract of PDB (300 mg/kg.d)	Same volume of saline	4 weeks	i.p.Alloxan (200 mg/kg)	11.1 mmol/L < FBG	FBG↓	*p* < 0.01
Qu et al. (2018)	Kunming mice	Gavage	NR	20 ∼ 23	Male	10/10	Triterpenes extract from PDB(200 mg/kg.d)	Same volume of saline	3 weeks	i.p.Alloxan (200 mg/kg)	11.1 mmol/L < FBG	1.Body weight↑; 2.FBG↓	1.*p* < 0.01; 2.*p* < 0.01
Shi et al. (2021)	SD rat	Gavage	NR	160 ∼ 200	Male	7/7	Flavonoids from PDB (160 mg/kg.d)	Same volume of saline	4 weeks	HFSD + i.p.STZ (30 mg/kg)	16.7 mmol/L < FBG	1.Body weight↑; 2.FBG↓; 3.FINS↓; 4.TG↓; 5.TC↓; 6.ISI↑	1.*p* < 0.05; 2.*p* < 0.05 3.*p* < 0.05; 4.*p* < 0.05 5.*p* < 0.05; 6.*p* < 0.05
Sun et al. (2010)	Wistar rat	Gavage	NR	180 ∼ 220	No restriction	8/8	Flavonoids from PDB (36 g/kg.d)	Same volume of saline	2 weeks	HFSD + i.p.STZ (50 mg/kg)	11.1 mmol/L < FBG	1.FBG↓; 2.FINS↓; 3.ISI↑; 4.SOD↑; 5.MDA↓	1.*p* < 0.05; 2.*p* < 0.05 3.*p* < 0.05; 4.*p* < 0.05 5.*p* < 0.05
Kong et al. (2021)	db/db Mice	Gavage	7	30 ∼ 40	Male	6/6	Flavonoids from PDB (400 mg/kg.d)	Same volume of distilled water	4 weeks	Spontaneous type 2 diabetic mellitus	-	1.FBG↓; 2.TG↓; 3.TC↓; 4.HDL-C↑; 5.LDL-C↓; 6.FINS↓; 7.IR↓; 8.SOD↑; 9.MDA↓	1.*p* < 0.05; 2.*p* < 0.05 3.*p* < 0.05; 4.*p* < 0.01 5.*p* > 0.05; 6.*p* < 0.01 7.*p* < 0.01; 8.*p* < 0.05 9.*p* < 0.05
Luo et al. (2020)	SD rat	Gavage	4	120 ∼ 140	Male	8/8	Aqueous extract of PDB (4 g/kg.d)	Same volume of saline	4 weeks	HFD + i.p.STZ (35 mg/kg)	16.7 mmol/L < RBG	1.Body weight↑; 2.TC↓; 3.TG↓; 4.HDL-C↓; 5.LDL-C↓	1.*p* > 0.05; 2.*p* < 0.05; 3.*p* > 0.05; 4.*p* > 0.05; 5.*p* < 0.05
Song et al. (2012)	Ob-db mice	Gavage	NR	20 ∼ 25	Male	8/8	Aqueous extract of PDB (2 g/kg·d)	Distilled water (0.5 mL/days)	4 weeks	HFD + i.p.Alloxan (60 mg/kg)twice	11.1 mmol/L ≤ FBG	1.Body weight↓; 2.FBG↓; 3.TC↓; 4.TG↓; 5.SOD↑; 6.MDA↓; 7.FFA↓	1.*p* > 0.05; 2.*p* < 0.01 3.*p* < 0.01; 4.*p* < 0.05 5.*p* < 0.05; 6.*p* < 0.05 7.*p* < 0.05
Su et al. (2016)	Wistar rat	Gavage	NR	195 ∼ 206	Male	10/10	Flavonoids from PDB (12 g/kg·d)	Same volume of distilled water	4 weeks	HFSD + i.p.STZ (40 mg/kg)	11.1 mmol/L ≤ FBG	1.FBG↓; 2.FINS↓; 3.ISI↑; 4.SOD↑; 5.MDA↓	1.*p* < 0.01; 2.*p* < 0.01 3.*p* < 0.01; 4.*p* < 0.01 5.*p* < 0.01
Tan et al. (2020)	SD rat	Gavage	NR	180 ∼ 220	Male	9/9	Flavonoids from PDB (216 mg/kg·d)	Saline (2 mL/kg)	8 weeks	HFSD + i.p.STZ (30 mg/kg)	11.1 mmol/L < FBG	1.Body weight↑; 2.FBG↓; 3.TC↓; 4.TG↓; 5.HDL-C↑; 6.LDL-C↓; 7.FFA↓	1.*p* < 0.05; 2.*p* < 0.05 3.*p* < 0.05; 4.*p* > 0.05 5.*p* < 0.05; 6.*p* < 0.05 7.*p* < 0.05

Abbreviations: CAT, catalase; FBG, fasting blood glucose; FFA, free fatty acid; FINS, fasting insulin; GSH-px, glutathione peroxidase; HDL-C, high-density lipoprotein cholesterol; HFD, high-fat diet; HFSD, high fat and sugar diet; I, intervention group; ICR, institute of cancer research; IR, insulin resistance; ISI, insulin sensitivity index; i.p., intraperitoneal; i.v., intravenous; LDL-C, low density lipoprotein cholesterol; MDA: malondialdehyde; M, model group; NO, nitric oxide; NOS, nitric oxide synthase; NR, not report; Ob-db, obese-diabetic; PBG, postprandial blood glucose; PDB, potentilla discolor bunge; RBG, random blood glucose; SD, sprague-dawley; STZ, streptozotocin; SOD, superoxide dismutase; TC, total cholesterol; TG, triglyceride.

### 3.3 Quality of the included studies

**FIGURE 2:**
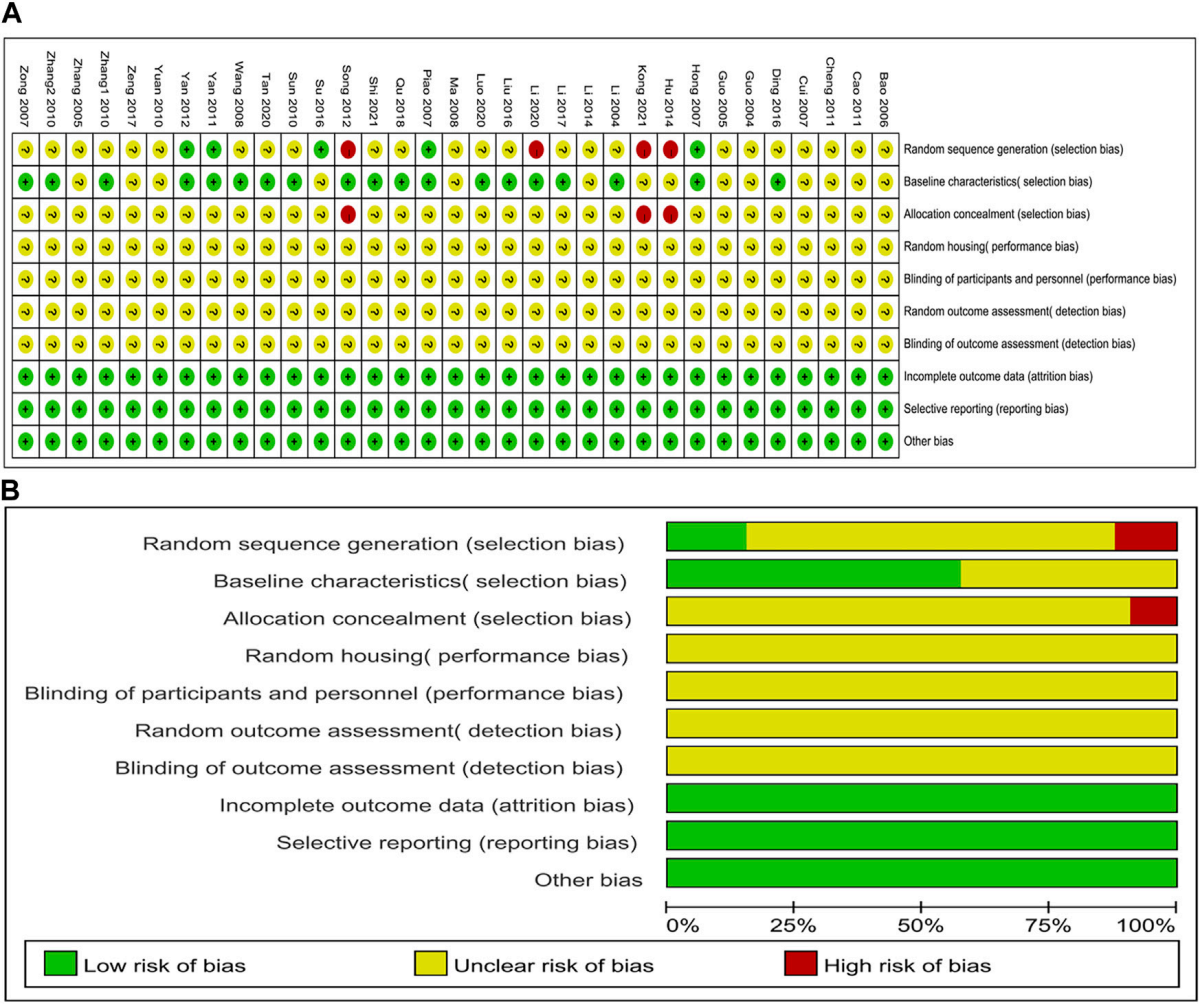
Evaluation of risk of bias. **(A)** summary for each risk of bias item for each study; **(B)** graph for each risk of bias item presented as percentages.

All studies were scored between 3 and 5 points; thirteen studies (Guo and Cui, 2004; Guo et al., 2005; Zhang and Shen, 2005; Bao et al., 2006; Cui et al., 2007; Ma and Cui, 2008; Yuan et al., 2010; Cao and Zhang, 2011; Cheng and Li, 2011; Hu et al., 2014; Li et al., 2014; Zeng et al., 2017; Kong et al., 2021) obtained three points; Fifteen studies (Li, 2004; Zong et al., 2007; Wang et al., 2008; Sun et al., 2010; Zhang et al., 2010; Song et al., 2012; Ding et al., 2016; Liu et al., 2016; Su et al., 2016; Li et al., 2017; Qu et al., 2018; Li et al., 2020; Luo et al., 2020; Tan et al., 2020; Shi, 2021) were scored four points; Four studies (Hong et al., 2007; Piao et al., 2007; Yan et al., 2011; Yan et al., 2012) received five points. For sequence generation, five studies (Hong et al., 2007; Piao et al., 2007; Yan et al., 2011; Yan et al., 2012; Su et al., 2016) were determined as low risk for using a random number table method, and four studies (Song et al., 2012; Hu et al., 2014; Li et al., 2020; Kong et al., 2021) that did not use randomization were considered high risk; The remaining studies only reported random assignments and did not specify a specific random sequence method; therefore, they were classified as having an unclear risk. Eighteen studies (Li, 2004; Hong et al., 2007; Piao et al., 2007; Zong et al., 2007; Wang et al., 2008; Sun et al., 2010; Zhang et al., 2010; Yan et al., 2011; Song et al., 2012; Yan et al., 2012; Ding et al., 2016; Liu et al., 2016; Li et al., 2017; Qu et al., 2018; Li et al., 2020; Luo et al., 2020; Tan et al., 2020; Shi, 2021) reported similar baseline characteristics between groups before the experiment and were identified as low risk. None of the studies clarified whether the assignment of different groups was adequately masked; none of the articles mentioned whether random housing and random outcome assessment were implemented; Similarly, none of the studies provided adequate details about the procedures used for blinding researchers, caregivers, and outcome measurement. Thus, all studies considered the risks of these items to be unclear. All studies reported expected outcomes, and none selectively reported data; hence, they were considered low risk. No study was identified as having a significant risk of additional bias. A thorough evaluation of the quality of the included studies is shown in Figure 2.

### 3.4 Results of meta-analyses

#### 3.4.1 Effect on fasting blood glucose (FBG)

**FIGURE 3:**
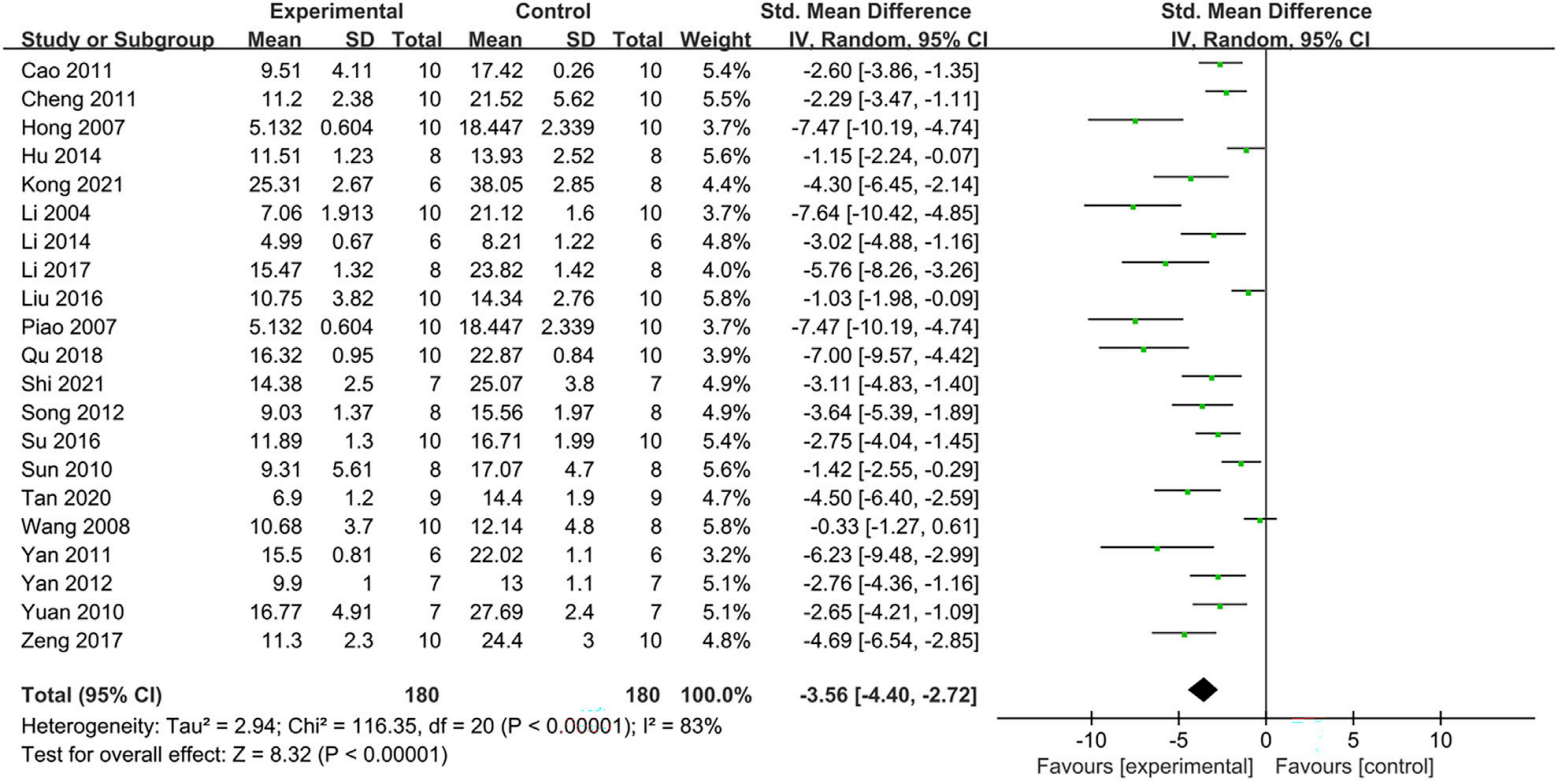
The effect of PDB extracts on FBG.

Twenty-one studies reported data on the effectiveness of PDB extracts on FBG levels. Their findings revealed that FBG level in the treatment group was considerably lower than that in the control group (SMD: −3.56 [95%CI: −4.40, −2.72], *p* < 0.00001; I^2^ = 83%, P_he_<0.00001) (Figure 3). Except for the results of two animal studies on Kunming mice, other subgroup analyses showed that PDB extracts significantly reduced FBG levels (Table 2).

**TABLE 2:** The results of subgroup analyses.

Parameter		Subgroup	Study (n)	Sample (n)	Effect estimate	*p*-Value	I^2^	P_heterogeneity_
**FBG**	Animal species	Wistar rat	9	164	−4.02 [−5.32, −2.72]	<0.0001	81%	<0.00001
		SD rat	5	88	−2.22 [−3.32, −1.12]	<0.001	72%	0.006
		Kunming mice	2	38	−3.55 [−10.08, 2.98]	0.29	96%	<0.00001
		db/dbmice (Spontaneous T2DM)	2	26	−4.89 [−6.68, −3.09]	<0.00001	0%	0.33
		C57BL/6 mice	1	12	−3.02 [−4.88, −1.16]	0.001	—	—
		ICR mice	1	16	−5.76 [−8.26, −3.26]	<0.00001	—	—
		Ob-db mice	1	16	−3.64 [−5.39, −1.89]	<0.0001	—	—
	Type of PDB	Flavonoids from PDB	10	172	−2.38 [−3.28, −1.47]	<0.00001	78%	<0.00001
		Aqueous extract of PDB	10	168	−4.47 [-5.65, −3.29]	<0.00001	72%	0.0002
		Triterpenes extract from PDB	1	20	−7.00 [−9.57, −4.42]	<0.00001	—	—
	Duration	2–4 weeks	16	274	−3.63 [−4.68, −2.59]	<0.00001	86%	<0.00001
		6–12 weeks	5	86	−3.33 [−4.42, −2.24]	<0.00001	55%	0.07
**FINS**	Animal species	Wistar rat	6	110	−1.14 [−2.62, 0.33]	0.13	88%	<0.00001
		SD rat	4	68	−1.35 [−1.89, −0.80]	<0.00001	0%	0.82
		db/db mice (Spontaneous T2DM)	2	24	−12.81 [−31.30, 5.69]	0.17	90%	0.001
		C57BL/6 mice	2	28	−0.14 [−5.86, 5.58]	0.96	96%	<0.00001
		Kunming mice	1	16	4.71 [2.59, 6.83]	<0.0001	-	-
	Type of PDB	Flavonoids from PDB	8	132	−1.41 [-2.65, −0.17]	0.03	85%	<0.00001
		Aqueous extract of PDB	7	114	−0.70 [−2.69, 1.28]	0.49	92%	<0.00001
	Duration	2–4 weeks	9	138	−1.21 [−3.05, 0.63]	0.20	91%	<0.00001
		6–12 weeks	6	108	−0.97 [−2.22, 0.29]	0.13	86%	<0.00001
**ISI**	Animal species	Wistar rat	5	90	2.24 [0.63, 3.86]	0.007	88%	<0.00001
		SD rat	1	20	1.36 [0.37, 2.36]	0.007	—	—
		db/dbmice (Spontaneous T2DM)	1	12	11.03 [5.53, 16.53]	<0.0001	—	—
	Type of PDB	Flavonoids from PDB	4	70	2.84 [0.66, 5.03]	0.01	90%	<0.00001
		Aqueous extract of PDB	3	52	2.37 [0.00, 4.73]	0.05	87%	<0.00001
	Duration	2–4 weeks	4	62	5.10 [1.25, 8.96]	0.009	92%	<0.00001
		6–12 weeks	3	60	1.11 [0.41, 1.81]	0.002	35%	0.21
**TG**	Animal species	Wistar rat	5	104	−1.31 [−2.07, −0.55]	0.0007	68%	0.008
		SD rat	6	98	−1.07 [−1.77, −0.36]	0.0003	60%	0.03
		db/dbmice (Spontaneous T2DM)	1	12	−1.27 [−2.56, 0.02]	0.05	—	—
		C57BL/6 mice	2	28	−4.96 [−6.67, −3.26]	<0.00001	0%	0.77
		Ob-db mice	1	16	−1.73 [−2.92, −0.53]	0.0005	—	—
	Type of PDB	Flavonoids from PDB	5	80	−0.69 [−1.15, −0.22]	0.004	0%	0.47
		Aqueous extract of PDB	10	168	−2.08 [−2.83, −1.33]	<0.00001	70%	0.0005
		Triterpenes extract from PDB	1	20	−0.37 [−1.25, 0.52]	0.42	—	—
	Duration	2–4 weeks	10	172	−1.29 [−1.85, −0.72]	<0.00001	64%	0.002
		6–12 weeks	5	86	−2.17 [−3.50, −0.85]	0.001	80%	0.0004
**TC**	Animal species	Wistar rat	5	104	−1.68 [−2.69, −0.67]	0.001	79%	0.0003
		SD rat	6	98	−2.00 [−3.11, −0.88]	0.0005	76%	0.0008
		db/dbmice (Spontaneous T2DM)	1	12	−3.45 [-5.47, −1.42]	0.0009	—	—
		C57BL/6 mice	2	28	−8.52 [−20.40, 3.36]	0.16	90%	0.002
		Ob/db mice	1	16	−2.52 [−3.92, −1.11]	0.0004	—	—
	Type of PDB	Flavonoids from PDB	5	80	−2.48 [-3.95, −1.02]	0.0009	80%	0.0005
		Aqueous extract of PDB	10	168	−2.32 [−3.24, −1.40]	<0.00001	77%	<0.0001
		Triterpenes extract from PDB	1	20	−0.36 [-1.25, 0.53]	0.43	—	—
	Duration	2–4 weeks	10	172	−2.07 [−2.94, −1.21]	<0.00001	79%	<0.00001
		6–12 weeks	5	86	−2.51 [−3.93, −1.09]	0.0005	78%	0.001
**HDL-C**	Animal species	Wistar rat	3	64	1.17 [0.16, 2.19]	0.02	72%	0.01
		SD rat	4	64	0.26 [-0.58, 1.10]	0.55	63%	0.05
		db/dbmice (Spontaneous T2DM)	1	12	2.01 [0.51, 3.50]	0.009	—	—
		C57BL/6 mice	2	28	2.63 [−0.19, 5.45]	0.07	79%	0.03
	Type of PDB	Flavonoids from PDB	4	66	0.95 [0.42, 1.48]	0.0004	1%	0.39
		Aqueous extract of PDB	6	92	1.34 [0.08, 2.60]	0.04	83%	<0.0001
		Triterpenes extract from PDB	1	20	0.25 [−0.63, 1.13]	0.001	—	—
	Duration	2–4 weeks	6	102	0.72 [−0.08, 1.52]	0.08	72%	0.001
		6–12 weeks	4	66	1.60 [0.65, 2.55]	0.0009	58%	0.07
**LDL-C**	Animal species	Wistar rat	3	64	−2.10 [−3.71, −0.50]	0.01	84%	0.0003
		SD rat	4	64	−1.23 [-2.02, −0.44]	0.002	49%	0.12
		db/dbmice (Spontaneous T2DM)	1	12	−1.04 [−2.29, 0.20]	0.10	—	—
		C57BL/6 mice	2	28	−5.25 [-10.67, 0.17]	0.06	83%	0.02
	Type of PDB	Flavonoids from PDB	4	66	−0.91 [−1.52, −0.31]	0.003	25%	0.26
		Aqueous extract of PDB	6	92	−3.17 [−4.63, −1.71]	<0.0001	77%	0.0006
		Triterpenes extract from PDB	1	20	−0.77 [−1.69, 0.14]	0.10	—	—
	Duration	2–4 weeks	6	102	−1.29 [-2.13, −0.44]	0.003	71%	0.002
		6–12 weeks	4	66	−2.74 [−4.14, −1.34]	<0.0001	78%	<0.0001
**MDA**	Animal species	Wistar rat	6	126	−3.95 [−5.75, −2.15]	<0.0001	90%	<0.00001
		SD rat	2	40	−2.71 [−3.61, −1.80]	<0.00001	0%	0.72
		db/dbmice (Spontaneous T2DM)	1	12	−6.76 [−10.24, −3.27]	0.0001	—	—
		Ob/db mice	1	16	−1.47 [−2.62, −0.33]	0.01	—	—
	Type of PDB	Flavonoids from PDB	6	108	−3.56 [-5.20, −1.92]	<0.0001	84%	<0.00001
		Aqueous extract of PDB	4	76	−4.16 [-6.40, −1.91]	0.0003	85%	0.0002
		Triterpenes extract from PDB	1	20	−0.86 [−1.78, 0.07]	0.07	—	—
	Duration	2–4 weeks	7	134	−3.05 [−4.38, −1.72]	<0.00001	85%	<0.00001
		6–12 weeks	3	60	−4.36 [−6.14, −2.59]	<0.00001	66%	0.05
**SOD**	Animal species	Wistar rat	6	126	2.89 [1.40, 4.39]	0.0001	89%	<0.00001
		SD rat	1	20	1.38 [0.38, 2.37]	0.007	—	—
		db/dbmice (Spontaneous T2DM)	1	12	5.62 [2.65, 8.58]	0.0002	—	—
		Ob/db mice	1	16	1.62 [0.45, 2.80]	0.007	—	—
	Type of PDB	Flavonoids from PDB	5	88	3.07 [1.24, 4.90]	0.001	87%	<0.00001
		Aqueous extract of PDB	4	76	3.08 [1.26, 4.89]	0.0009	85%	0.0002
		Triterpenes extract from PDB	1	20	0.34 [−0.54, 1.23]	0.45	—	—
	Duration	2–4 weeks	7	134	2.98 [1.56, 4.41]	<0.0001	88%	<0.00001
		6–12 weeks	2	40	2.05 [1.25, 2.85]	<0.00001	0%	0.43
**GSH-px**	Animal species	Wistar rat	2	50	0.83 [−0.10, 1.75]	0.08	65%	0.06
		SD rat	2	40	1.60 [0.75, 2.45]	0.0002	24%	0.25
	Type Of PDB	Aqueous extract of PDB	1	20	1.90 [0.81, 3.00]	0.0007	—	—
		Flavonoids from PDB	3	60	1.19 [0.27, 2.11]	0.01	61%	0.08
		Triterpenes extract from PDB	1	20	0.31 [−0.57, 1.19]	0.49	—	—
	Duration	2–4 weeks	2	50	0.88 [−0.14, 1.91]	0.09	71%	0.03
		6–12 weeks	2	40	1.52 [0.79, 2.24]	<0.0001	0%	0.46
**FFA**	Animal species	Wistar rat	1	14	−5.92 [−8.71, −3.13]	<0.0001	—	—
		SD rat	2	34	−0.97 [-2.62, 0.68]	0.25	79%	0.03
		Ob/db mice	1	16	−1.96 [−3.22, −0.71]	0.002	—	—
		C57BL/6 mice	1	16	−11.20 [−15.78, −6.63]	<0.00001	—	—
	Type of PDB	Flavonoids from PDB	2	34	−0.97 [-2.62, 0.68]	0.25	79%	0.03
		Aqueous extract of PDB	3	46	−5.94 [-10.70, −1.18]	0.01	90%	<0.0001
	Duration	2–4 weeks	3	46	−2.33 [−4.79, 0.13]	0.06	88%	0.0002
		6–12 weeks	2	34	−6.25 [−15.41, 2.92]	0.18	93%	0.0001
**Body weight**	Animal species	Wistar rat	3	58	1.91 [0.05, 3.76]	0.04	88%	<0.00001
		SD rat	5	78	0.64 [0.10, 1.18]	0.02	25%	0.25
		C57BL/6 mice	2	28	−0.22 [−9.51, 9.07]	0.96	97%	<0.00001
		Kunming mice	2	38	2.79 [1.84, 3.74]	<0.00001	0%	0.53
		Ob/db mice	1	16	−0.01 [−0.99, 0.97]	0.99	—	—
	Type of PDB	Flavonoids from PDB	5	86	1.08 [0.32, 1.85]	0.006	61%	0.04
		Aqueous extract of PDB	7	102	1.19 [−0.50, 2.88]	0.17	89%	<0.00001
		Triterpenes extract from PDB	2	40	1.54 [−1.44, 4.52]	0.31	92%	0.0003
	Duration	2–4 weeks	10	172	1.22 [0.45, 1.98]	0.002	79%	<0.00001
		6–12 weeks	3	46	0.45 [−3.77, 4.66]	0.83	93%	<0.00001

#### 3.4.2 Effect on fasting insulin (FINS)

**FIGURE 4:**
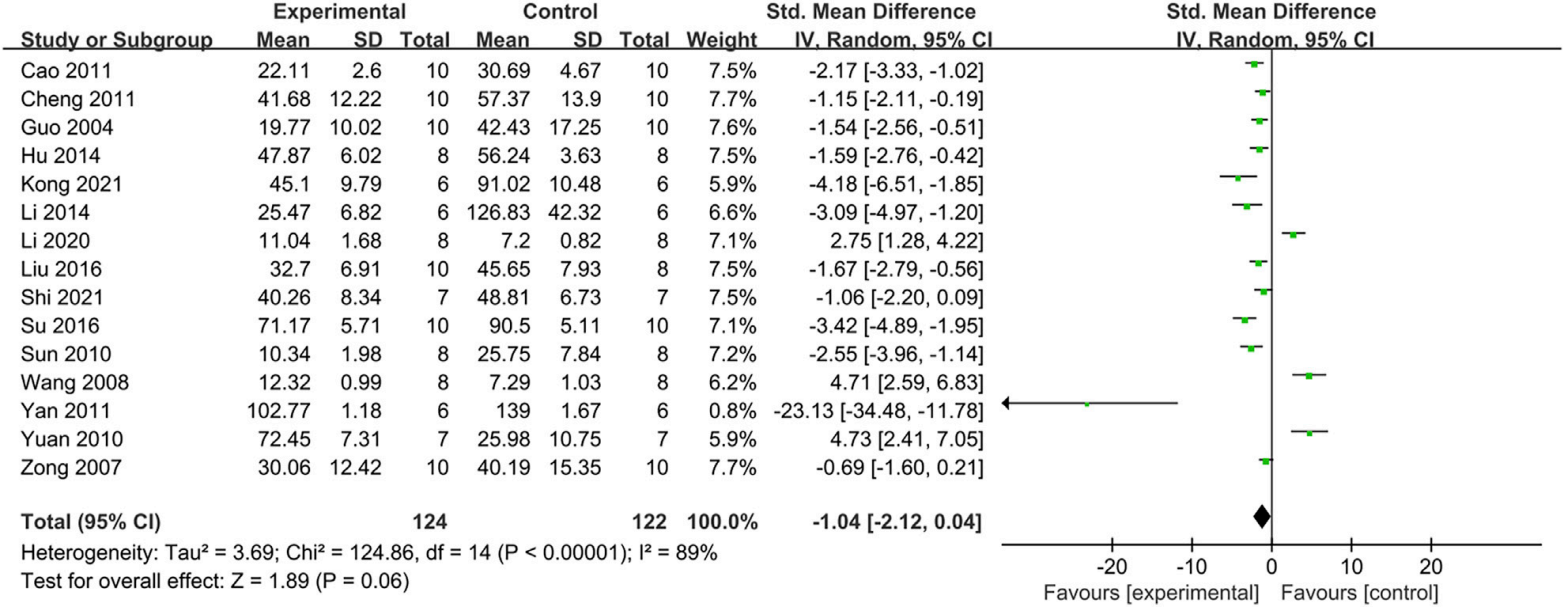
The effect of PDB extracts on FINS.

For comparing FINS, data from fifteen animal studies were combined. There was no significant difference between the intervention group and the control group (SMD: −1.04, [95%CI: −2.12, 0.04], *p* = 0.06; I^2^ = 89%, P_he_<0.00001) (Figure 4). In subgroup analyses, results from SD rats showed that PDB extracts suppressed insulin secretion (SMD: −1.35, [95%CI: −1.89, −0.80], *p* < 0.00001; I^2^ = 0%, P_he_ = 0.82). Eight studies of flavonoids from PDB obtained similar results. Interestingly, one study on Kunming mice showed that PDB extracts increased insulin secretion (Table 2).

#### 3.4.3 Effect on insulin sensitivity index (ISI)

**FIGURE 5:**
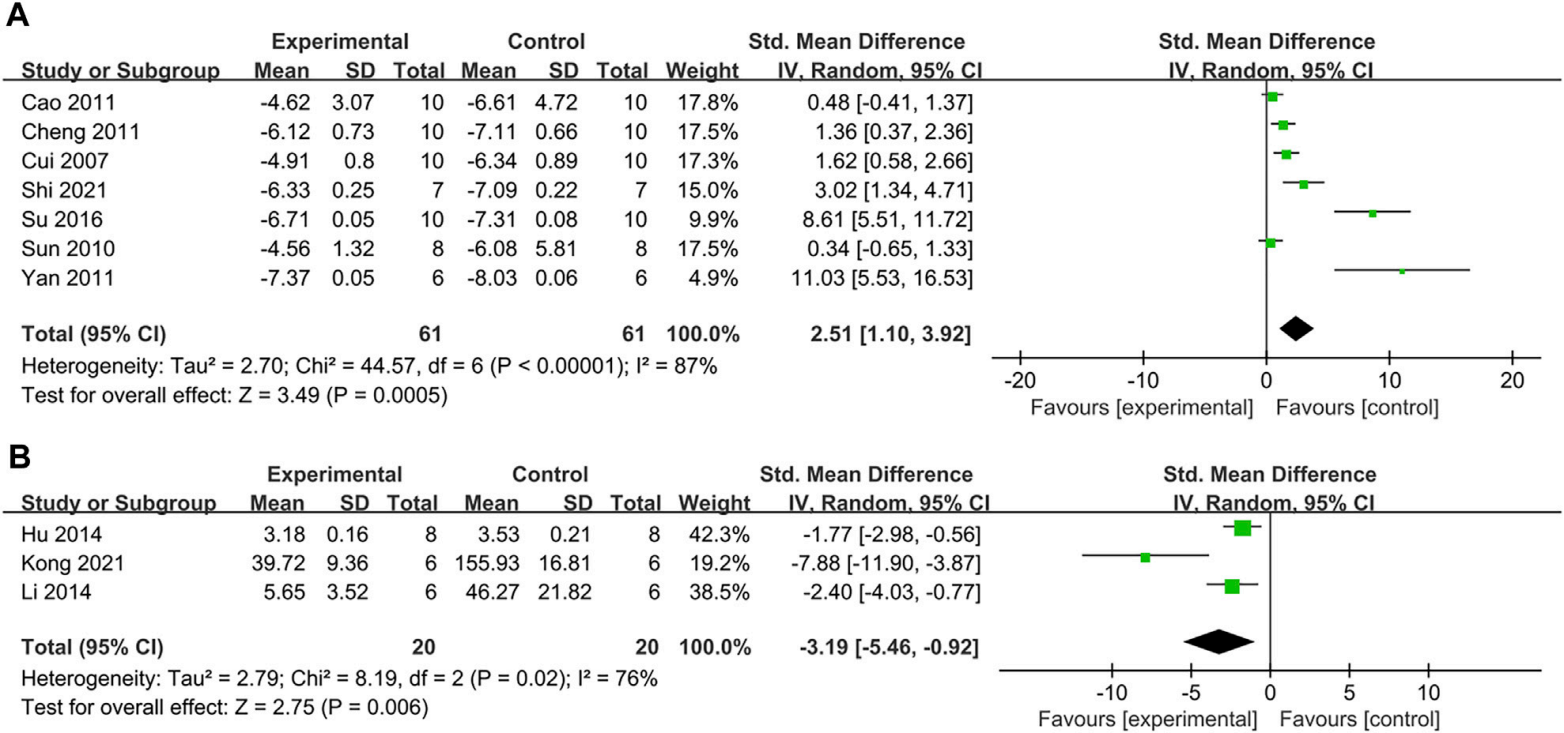
**(A)** The effect of PDB extracts on ISI; **(B)** The effect of PDB extracts on IR.

The effect of PDB extracts on ISI was reported in seven trials. The overall analysis demonstrated that PDB extracts improved ISI in animal models of diabetes (SMD: 2.51 [95% CI: 1.10, 3.92], *p* = 0.0005; I^2^ = 87%, P_he_ < 0.00001) (Figure 5A). The subgroup analyses were consistent with the overall results, except the results of three studies that used the aqueous extracts of PDB. The results of three studies showed no significant difference between the control and treatment groups (Table 2).

#### 3.4.4 Effect on insulin resistance (IR)

Three studies reported the effectiveness of PDB extracts on IR. As shown in Figure 5B, the pooled results showed that compared with the control group, PDB extracts significantly decreased the IR (SMD: −3.19 [95% CI: −5.46, −0.92], *p* = 0.006; I^2^ = 76%, P_he_ = 0.02).

#### 3.4.5 Effect on triglyceride (TG)

**FIGURE 6:**
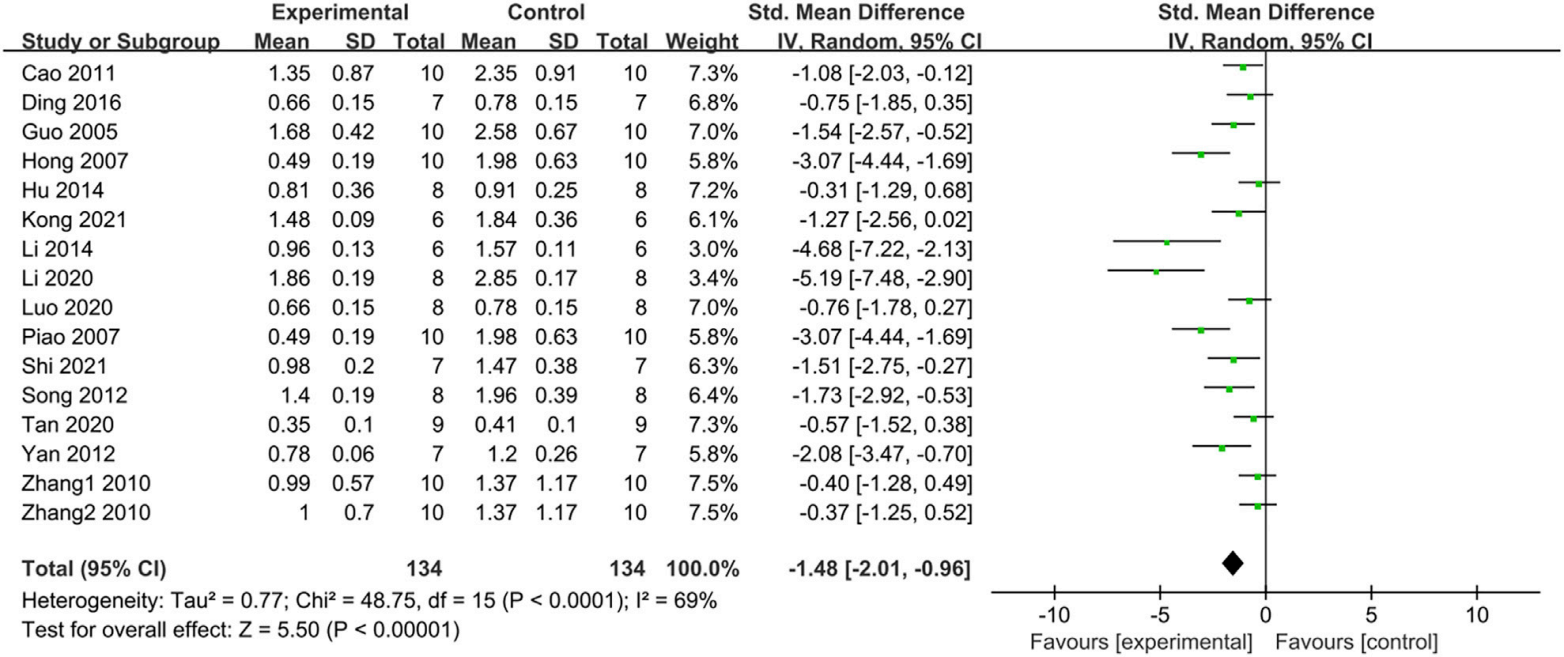
The effect of PDB extracts on TG.

The pooled results of fifteen studies with 16 groups indicated that PDB extracts significantly decreased TG levels (SMD: −1.48, [95% CI: −2.01, −0.96], *p* < 0.00001; I^2^ = 69%, P_he_ <0.0001) (Figure 6). Subgroup analyses also showed significant reductions in TG levels with PDB extracts, except one study on spontaneous T2DM and one study on PDB-derived triterpenes (Table 2).

#### 3.4.6 Effect on total cholesterol (TC)

**FIGURE 7:**
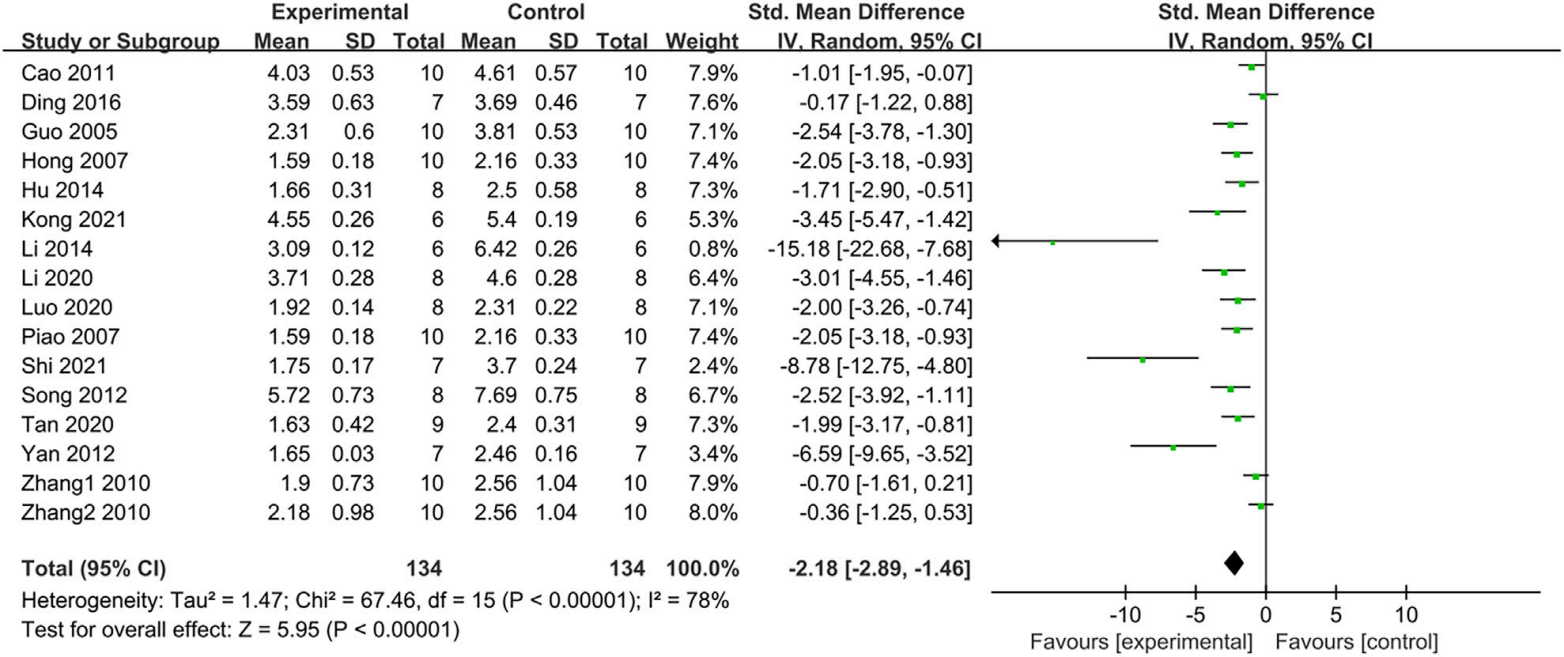
The effect of PDB extracts on TC.

Fifteen studies with 16 groups explored the effects of PDB extracts on TC. The combined data showed that the TC level in the intervention group was much lower than in the control group (SMD: −2.18, [95%CI: −2.89, −1.46], *p* < 0.00001; I^2^ = 78%, P_he_<0.00001) (Figure 7). However, the subgroup analysis of two studies on C57BL/6 mice showed no significant difference in TC level between the treatment group and the control group. A study with PDB-derived triterpenes showed similar results. The remaining subgroup analyses were consistent with the overall results (Table 2).

#### 3.4.7 Effect on high-density lipoprotein cholesterol (HDL-C)

**FIGURE 8:**
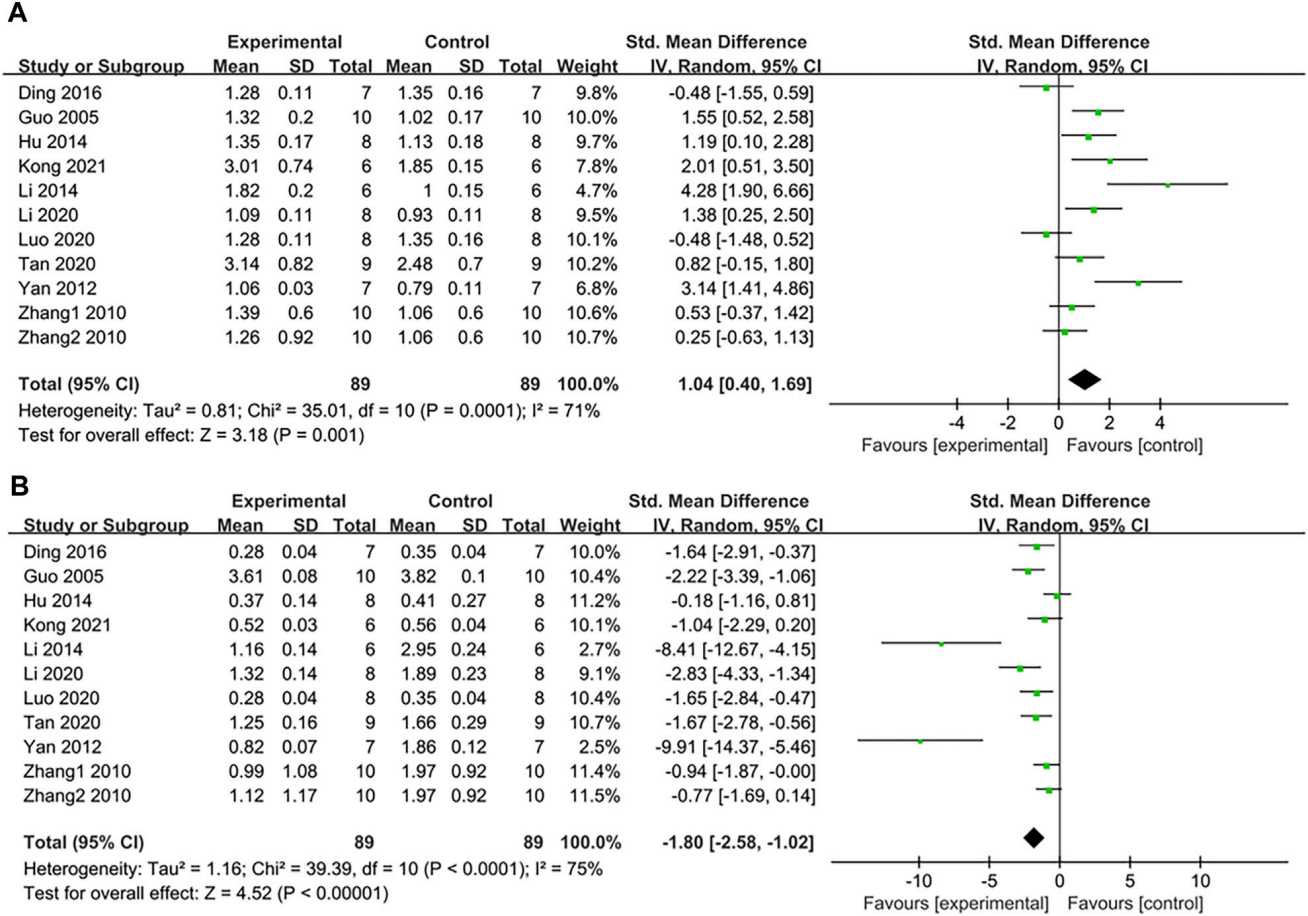
**(A)** The effect of PDB extracts on HDL-C; **(B)** The effect of PDB extracts on LDL-C.

The pooled data from 10 studies with 11 groups suggested that PDB extracts significantly increased HDL-C levels compared to the control group (SMD: 1.04, [95% CI: 0.40, 1.69], *p* = 0.001; I^2^ = 71%, P_he_ = 0.0001) (Figure 8A). Nevertheless, subgroup analyses of studies on SD rats and C57BL/6 mice, studies using aqueous extracts of PDB, and studies with treatment duration of shorter than 6 weeks indicated that there was no significant difference in HDL levels between the treatment group and the control group (Table 2).

#### 3.4.8 Effect on low-density lipoprotein cholesterol (LDL-C)

The effect of PDB extracts on LDL-C levels was examined in ten studies with eleven groups. Compared to the control group, PDB extracts significantly lowered LDL-C levels (SMD: −1.80, [95% CI: −2.58, −1.02], *p* < 0.00001; I^2^ = 75%, P_he_<0.0001) (Figure 8B). Except for the results of two studies on C57BL/6 mice, one study on spontaneous T2DM mice, and one study on PDB-derived triterpenes extracts, other subgroup analyses showed that PDB extracts significantly reduced LDL-C levels (Table 2).

#### 3.4.9 Effect on malondialdehyde (MDA)

**FIGURE 9:**
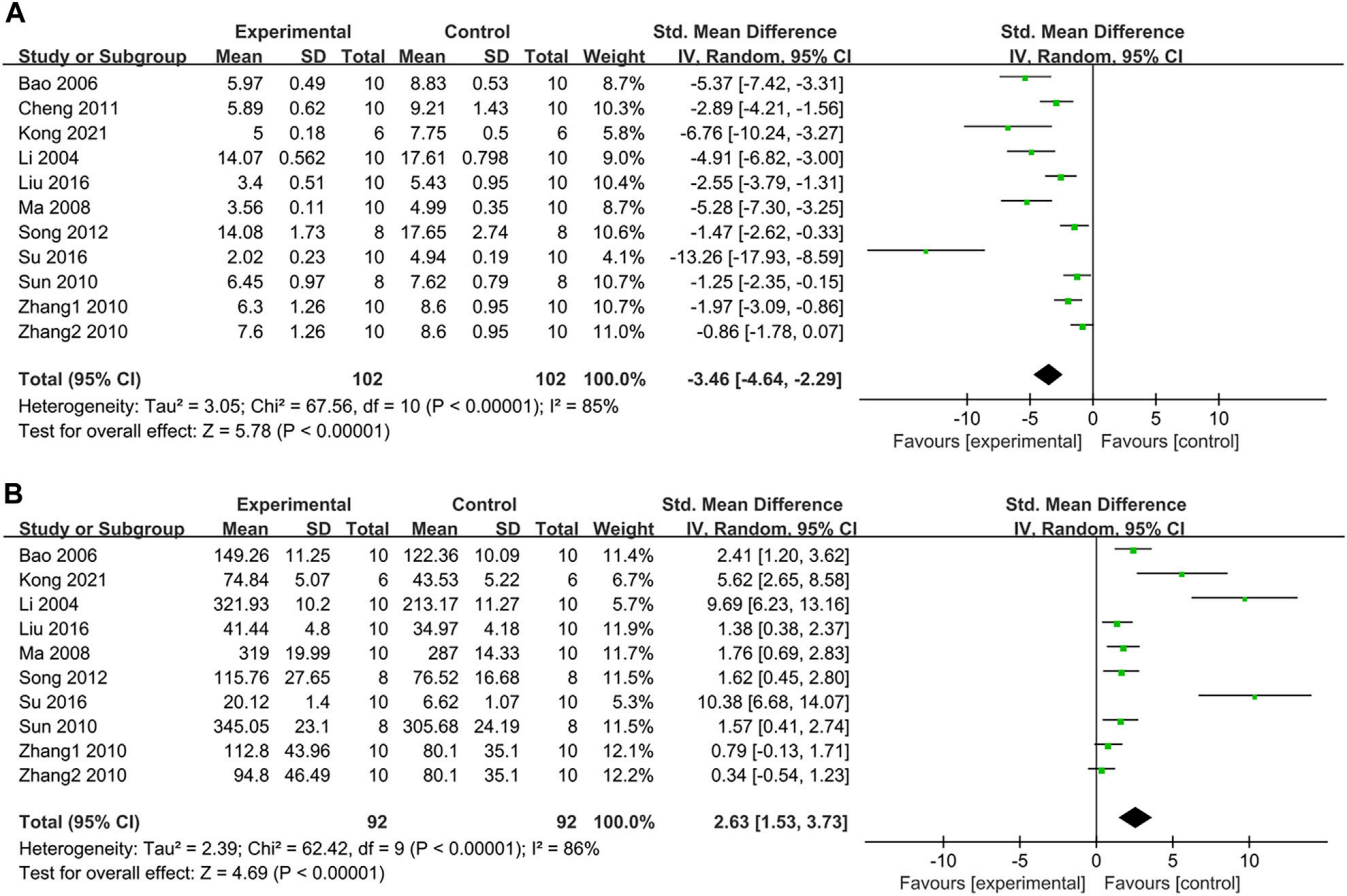
**(A)** The effect of PDB extracts on MDA; **(B)** The effect of PDB extracts on SOD.

As shown in Figure 9A, the pooled result of ten studies with 11 groups exhibited that PDB extracts effectively decreased MDA levels compared to the control group (SMD: −3.46, [95% CI: −4.64, −2.29], *p* < 0.00001; I^2^ = 85%, P_he_<0.00001). Except one study that used PDB-derived triterpenes, all subgroup analyses supported the overall results (Table 2).

#### 3.4.10 Effect on superoxide dismutase (SOD)

SOD level was assessed in nine studies with ten groups. The results indicated that PDB extracts significantly increased SOD levels (SMD: 2.63, [95% CI: 1.53, 3.73], *p* < 0.00001; I^2^ = 86%, P_he_ < 0.00001) (Figure 9B). All subgroup analyses showed similar results except one study with PDB-derived triterpenes (Table 2).

#### 3.4.11 Effect on glutathione peroxidase (GSH-px)

**FIGURE 10:**
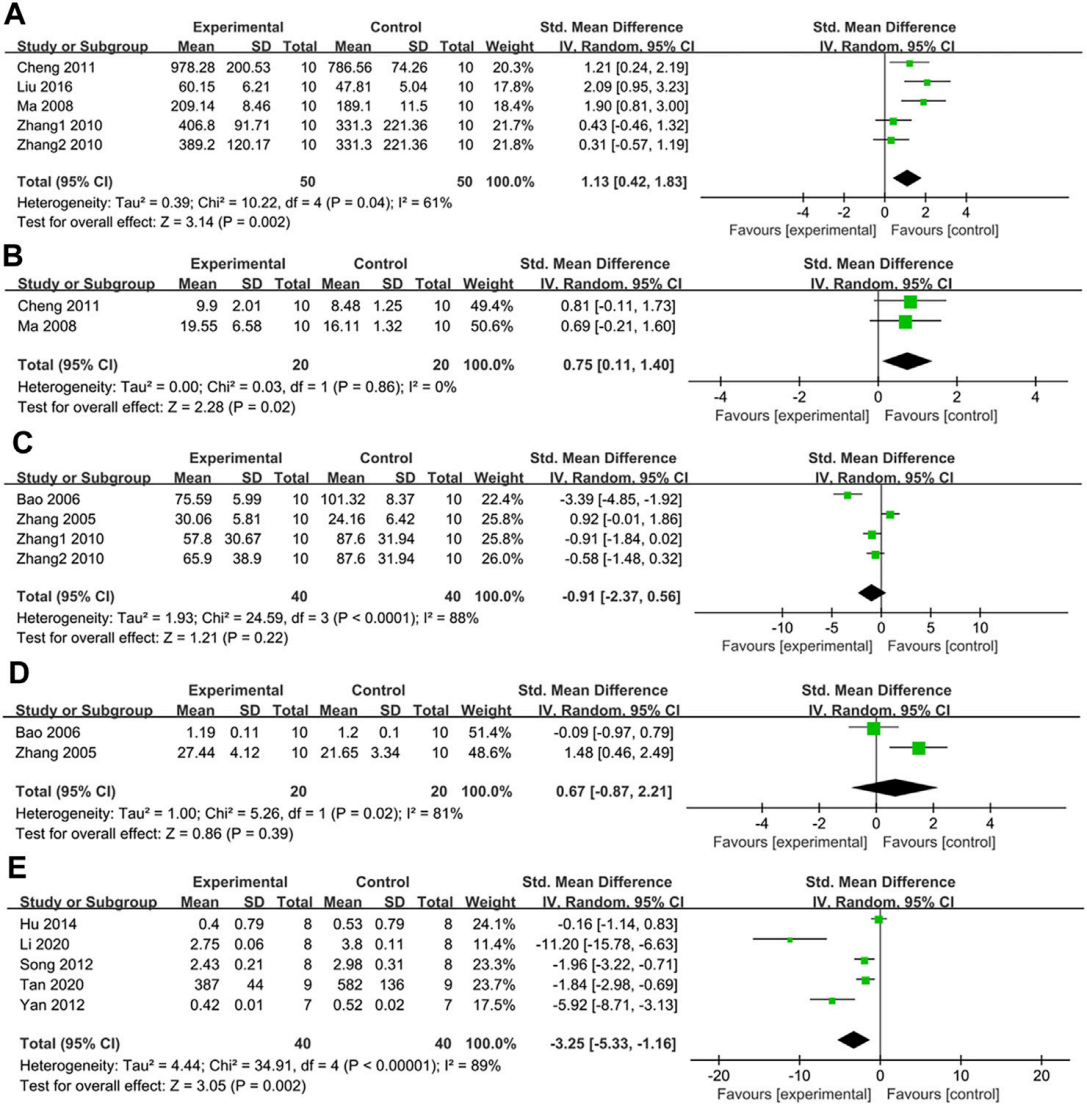
**(A)** The effect of PDB extracts on GSH-px; **(B)** The effect of PDB extracts on CAT; **(C)** The effect of PDB extracts on NO; **(D)** The effect of PDB extracts on NOS; **(E)** The effect of PDB extracts on FFA.

In four studies with five groups, GSH-px was measured. Their findings revealed that GSH-px levels were higher in the treatment group than in the control group (SMD: 1.13, [95% CI: 0.42, 1.83], *p* = 0.002; I^2^ = 61%, Phe = 0.04) (Figure 10A). All subgroup analyses showed similar results except one study with PDB-derived triterpenes and two studies on Wistar rats (Table 2).

#### 3.4.12 Effect on catalase (CAT)

Two studies assessed the effect of PDB extracts on CAT. Their results showed that CAT levels were higher in the treatment group than in the control group (SMD: 0.75, [95% CI: 0.11, 1.40], *p* = 0.02; I^2^ = 0%, Phe = 0.86) (Figure 10B).

#### 3.4.13 Effect on nitric oxide (NO)

Three studies with four groups measured the effect of PDB extracts on NO levels. There was no significant difference between the treatment group and the control group according to the combined results (SMD: −0.91, [95% CI: −2.37, 0.56], *p* = 0.22; I^2^ = 88%, P_he_<0.0001) (Figure 10C).

#### 3.4.14 Effect on nitric oxide synthase (NOS)

Two studies compared NOS levels between the treatment group and the control group. The pooled results showed that there was no significant difference in NOS levels between the two groups (SMD: 0.67, [95% CI: −0.87, 2.21], *p* = 0.39; I^2^ = 81%, P_he_ = 0.02) (Figure 10D).

#### 3.4.15 Effect on free fatty acid (FFA)

Five studies reported the effect of PDB extracts on FFA. The combined results showed that FFA levels were considerably lower in the treatment group (SMD: −3.25, [95% CI: −5.33, −1.16], *p* = 0.002; I^2^ = 89%, P_he_<0.00001) (Figure 10E). Subgroup analysis revealed no significant difference in FFA levels between the intervention group and the control group in studies on SD rats treated with PDB-derived flavonoids. Surprisingly, subgroup analyses based on the duration of treatment revealed that PDB extracts had no discernible impact on FFA compared to the control group (Table 2).

#### 3.4.16 Effect on body weight

**FIGURE 11:**
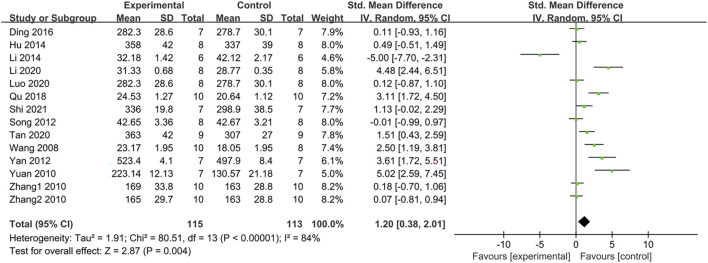
The effect of PDB extracts on body weight.

Body weight was assessed in 13 studies with 14 groups. Compared with the control group, PDB extracts significantly accelerated weight gain (SMD: 1.20, [95% CI: 0.38, 2.01], *p* = 0.004; I^2^ = 84%, P_he_<0.00001) (Figure 11). Subgroup analyses based on animal species showed no significant difference in body weight between the intervention and control groups in C57BL/6 mice and Ob/db mice. Studies with treatment durations longer than 6 weeks revealed similar results. Subgroup analysis based on the components of PDB revealed that only PDB-derived flavonoids increased body weight compared to the control group (Table 2).

### 3.5 Results of meta-regression

In order to better find significant influencing factors affecting the heterogeneity of results, meta-regression analysis was performed on indicators containing 10 or more studies based on animal species, type of PDB extracts, and treatment duration. As presented in Table 3, the results of regression analysis showed that the constituent of PDB extracts was a significant factor responsible for the heterogeneity of the effect of PDB extracts on TG (*p* = 0.04). The remaining indicators showed that heterogeneity was not significantly associated with the constituent of PDB extracts, treatment duration, and animal species. The results of meta-regression were almost consistent with those of subgroup analyses.

**TABLE 3:** The results of meta-regression analyses.

Parameter	Variable	Coefficient	Std. Err	*t*	*p*-Value	95% CI
FBG	Type of PDB	0.491	0.701	0.70	0.493	−0.977, 1.958
	Duration	0.044	0.205	0.22	0.832	−0.385, 0.473
	Animal species	−0.005	0.254	−0.02	0.984	−0.537, 0.5278
FINS	Type of PDB	−0.479	2.147	−0.22	0.827	−5.116, 4.159
	Duration	−0.029	0.382	−0.08	0.940	−0.855, 0.796
	Animal species	0.324	0.807	0.40	0.694	−1.419, 2.068
TG	Type of PDB	0.989	0.424	2.33	0.04	0.080, 1.899
	Duration	−0.203	0.151	−1.35	0.20	−0.527, 0.119
	Animal species	−0.228	0.291	−0.78	0.45	−0.853, 0.397
TC	Type of PDB	0.566	0.910	0.62	0.54	−1.386, 2.518
	Duration	−.192	0.270	−0.71	0.49	−0.772, 0.387
	Animal species	−0.479	0.463	−1.04	0.32	−1.471, 0.513
HDL-C	Type of PDB	−0.389	0.606	−0.64	0.54	−1.760, 0.982
	Duration	0.209	0.160	1.31	0.22	−0.153, 0.571
	Animal species	0.705	0.311	2.27	0.05	0.002, 1.409
LDL-C	Type of PDB	1.072	0.624	1.72	0.12	−0.339, 2.484
	Duration	−0.265	0.177	−1.50	0.17	−0.665, 0.135
	Animal species	−.744	0.608	−1.22	0.25	−2.119, 0.631
Body weight	Type of PDB	0.126	0.900	0.14	0.89	−1.835, 2.087
	Duration	−0.116	0.320	−0.36	0.72	−0.813, 0.582
	Animal species	0.262	0.305	0.86	0.41	−0.402, 0.927
SOD	Type of PDB	−1.051	1.767	−0.60	0.57	−5.127, 3.024
	Duration	−0.010	0.542	−0.02	0.99	−1.260, 1.239
	Animal species	−0.634	1.107	−0.57	0.58	−3.187, 1.919
MDA	Type of PDB	1.144	1.525	0.75	0.47	−2.307, 4.594
	Duration	−0.181	0.315	−0.57	0.58	−0.894, 0.532
	Animal species	0.476	1.011	0.47	0.65	−1.812, 2.764

### 3.6 Sensitivity analysis

Sensitivity analyses were performed to investigate the stability of the results. After excluding the studies one by one, the results revealed that the effects of PDB extracts on FINS were unstable. Three studies showed that PDB extracts increased insulin secretion and significantly affected the results of FINS. After removing these articles one by one, the combined results were reversed and showed that PDB and its extracts significantly reduced FINS in animal models of diabetes (Supplementary Material S3). After sensitivity analyses, the remaining results remained unchanged, proving that the findings were stable. The details of all sensitivity analyses are shown in Supplementary Material S3.

### 3.7 Publication bias

Nine outcomes were measured in ten or more studies, including FBG, MDA, HDL-C, FINS, TC, TG, LDL-C, SOD, and body weight. Hence, Egger’s test and funnel plot were performed. No publication bias was found regarding the impact of PDB extracts on FINS (*p* = 0.899) and body weight (*p* = 0.108). However, potential publication bias existed regarding FBG (*p* < 0.001), TG (*p* < 0.001), TC (*p* < 0.001), HDL-C (*p* < 0 .001), LDL-C (*p* < 0.001), MDA (*p* < 0.001), and SOD (*p* < 0.001). Details are provided in Supplementary Material S4. Then, the trim-and-fill method was used to determine the impact of publication bias on the outcomes. Trim-and-fill methods for FBG, TG, TC, LDL-C, MDA, and SOD showed no trimming, and the results were unchanged. Regarding HDL-C, the result was also not reversed after filling in one study. Hence, the findings of this meta-analysis were all robust. Detailed results of the trim-and-fill method are provided in Supplementary Material S5.

## 4 Discussion

To our knowledge, this is the first preclinical systematic review and meta-analysis that evaluated the efficacy of PDB extracts on oxidative stress and glycolipid metabolism in animal models of diabetes. In total, 32 studies with 574 animals were included, and 16 outcomes were analyzed. DM is characterized by chronic hyperglycemia and is closely related to dyslipidemia. Our study demonstrated that the PDB extracts increased HDL-C levels while decreasing TC, TG, LDL-C, and FBG levels. Although there was publication bias, the results did not change after the trim-and-fill method, suggesting their reliability. Additionally, the sensitivity analysis indicated that the outcomes were stable. These demonstrated that the extracts of PDB have great potential in improving glycolipid metabolism. Overall pooled data revealed no discernible difference in FINS between the control and treatment groups. Sensitivity analysis suggested that after removing three studies, the combined results showed that PDB extracts reduced insulin secretion. The findings of these three studies demonstrated that PDB extracts enhanced insulin secretion. After careful consideration, these articles were not removed because, as a compensatory mechanism, FINS increases or decreases in different stages of DM. When insulin resistance is dominant, FINS shows a compensatory increase; insulin secretion decreases when the islet β-cell is damaged. In this study, the methods of animal modeling were different. Some animals were given single or double injections of Alloxan or STZ, resembling type 1 diabetes, in which β cell damage is dominant. (Syed, 2022). Under this situation, PDB extracts promoted the repair of β cells and increased insulin secretion. Some animals were fed a high-fat diet and received low doses of STZ or alloxan, resembling the T2DM model, characterized by insulin resistance and partial dysfunction of β cells. (Stumvoll et al., 2005). In this case, PDB extracts reduced insulin resistance and increased insulin sensitivity, thus reducing the secretion of FINS. Therefore, the increase and decrease in FINS after using PDB extracts are not contradictory. Weight loss is a typical symptom of DM. According to our study, PDB extracts can dramatically enhance body weight. PDB was shown to improve the diversity, composition, and structure of intestinal flora in rats with T2DM, which may affect body weight (He S. Y. et al., 2022). Nevertheless, more studies are needed to uncover the precise mechanism.

**FIGURE 12:**
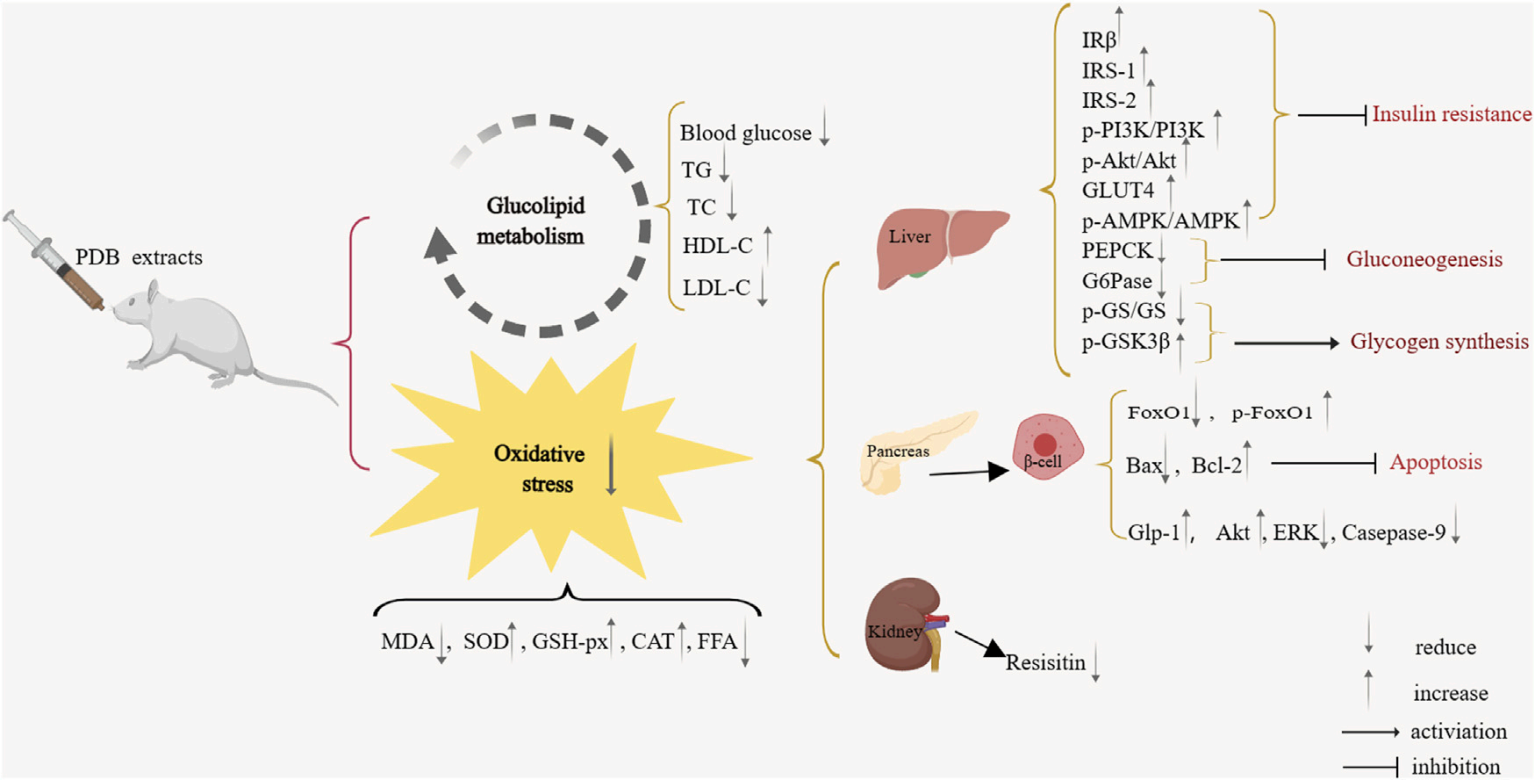
The mechanism of PDB extracts in ameliorating diabetes mellitus.

Oxidative stress, a significant contributor to DM, is caused by increased production of reactive oxygen species (ROS), decreased levels of endogenous antioxidants, or both (Zhang et al., 2020). In normal conditions, endogenous antioxidants such as SOD, GSH-px, and CAT can protect against oxidative stress (Banerjee and Vats, 2014; Mansuroğlu et al., 2015). Hyperglycemia and FFA can lead to ROS overproduction (Inoguchi et al., 2000; Zhang et al., 2020); Uncontrolled oxidative stress can increase MDA levels (Del Rio et al., 2005), causing insulin resistance and β cell malfunction (Zhang et al., 2020). This study showed that PDB extracts could considerably lower MDA and FFA levels while raising SOD, GSH-px, and CAT levels. These findings indicate that the PDB extracts possess obvious antioxidant properties. According to the existing literature, some studies have explored the specific mechanisms by which PDB meliorates DM. Most of these studies showed that PDB reduces oxidative stress. PDB extracts also activated protein kinase B (Akt) and AMP-activated protein kinase (AMPK) and inhibited phosphoenolpyruvate carboxykinase (PEPCK) and glucose-6-phosphatase (G6Pase) expression in the liver to prevent hepatic gluconeogenesis, decrease glycogen synthase (GS) phosphorylation and enhance glycogen synthase kinase 3β (GSK3β) phosphorylation to promote glycogen synthesis (Li et al., 2020). Furthermore, PDB extracts promoted the expression of insulin receptor β (IRβ), insulin receptor substrate-1 (IRS-1), insulin receptor substrate-2 (IRS-2), and glucose transporter-4 (GLUT4) in the liver to enhance insulin sensitivity and reduce insulin resistance (Hu et al., 2014; Kong et al., 2021). One study showed that PDB extracts upregulated the expression of glucagon-like peptide-1 (GLP-1) and Akt and downregulated the expression of the extracellular signal-regulated kinase (ERK) and caspase-9 to protect β cells (Tan et al., 2020). Besides, PDB extracts reduced the expression of Bax and increased the expression of Bcl-2 to inhibit the apoptosis of β cells (Liu et al., 2016). In addition, decreasing the expression of FOXO1 and increasing the expression of p-FoxO1 are other mechanisms by which PDB protects β cells (Ding et al., 2016; Luo et al., 2020). Only one study investigated the effects of PDB extracts on the kidney, showing that PDB significantly reduced the mRNA expression of resistin in perirenal adipose tissue of rats to reduce insulin resistance (Zong et al., 2007). These mechanisms are shown in Figure 12. Except for ameliorating oxidative stress, whether PDB extracts improve DM by reducing inflammation is still unknown. More studies should be conducted in the future.

This systematic review also has its limitations. Firstly, most of the included studies were published in Chinese, which may lead to language bias. Second, the risk of bias evaluation revealed that the majority of studies did not report the baseline data of the animals, the method of randomization, and whether blinding was used for caregivers and assessors; therefore, a majority of studies scored three to four points out of 10, this may reduce the credibility of the results. Third, there was a lack of data on the adverse effects of PDB extracts. The absence of safety data hampered our evaluation of the long-term tolerability of PDB extracts. Fourth, although subgroup analyses and meta-regression were performed according to animal species, constituents of PDB extracts, and duration of treatment, heterogeneity did not significantly decrease, which may be due to other factors, such as modeling and statistical methods. Thus, it is difficult to find the most prominent factors affecting heterogeneity. Therefore, the random-effects model was used to avoid the influence of heterogeneity on the results as much as possible. Fifth, although 32 studies were included in this systematic review, not all disclosed desired outcomes. Therefore, some indicators, such as NO and NOS, may have been misinterpreted due to the limited number of articles.

The findings of this study may have some implications for future research. The extracts discussed in this systematic review included aqueous extracts, flavonoids, and triterpenes. The aqueous extracts are provided by concentrating the liquid after boiling PDB. Its components are undoubtedly more complex than flavonoids and triterpenes. Flavonoids are found in almost all parts of PDB, constituting approximately 20% of PDB extract, with many pharmacological and physiological activities (Fu, 2017; Qin et al., 2020). Subgroup analyses showed that flavonoids and aqueous extracts were equally effective on most indicators. Therefore, we speculated that PDB ameliorates DM primarily through flavonoids. Although, further studies are needed to verify this hypothesis. Hong *et al.* found seven flavonoid monomers in PDB (Hong et al., 2013). Li et al. found six triterpenoid monomers in PDB (Li et al., 2013). Previous studies have roughly verified the anti-diabetic efficacy of PDB extracts. Further studies are needed to determine which monomer is the most effective for DM. In addition, whether PDB extracts exert the same therapeutic effects in the human body remains to be verified with well-designed RCTs in the future.

## 5 Conclusion

Current evidence suggests that PDB extracts can improve glycolipid metabolism and oxidative stress, alleviate pancreatic β-cell injury, reduce insulin resistance, and increase insulin sensitivity in diabetic animals. Taken together, the results suggested that PDB might be a promising medication for treating DM. However, the findings should be interpreted with caution due to the significant heterogeneity between studies and the low-to-moderate quality of studies. In addition, it is necessary to investigate which constituent of PDB plays the major role in ameliorating DM and whether these benefits can be reproduced in the human body.

## Data Availability

The original contributions presented in the study are included in the article/Supplementary Material, further inquiries can be directed to the corresponding author.
