# Supplementary material for: Effects of potentilla discolor bunge extracts on oxidative stress and glycolipid metabolism in animal models of diabetes: a systematic review and meta-analysis
Source: Front Pharmacol. 2023 Oct 2;14:1218757. doi: 10.3389/fphar.2023.1218757 (PMC10577192; doi:10.3389/fphar.2023.1218757)
Supplement: Supplementary file 2 [file Table2.DOCX]

***Supplementary Material 2******-Search strategy of Pubmed***

**Effects of Potentilla discolor Bunge extracts on oxidative stress and glycolipid metabolism in diabetic animal models: A systematic review and meta-analysis**

Yunjiao Yang, Wen Deng, Yue Wu, Changyan Zi, Qiu Chen^,^*

***Corresponding author:** Qiu Chen

E-mail: [chenqiu1005@cdutcm.edu.cn](mailto:chenqiu1005@cdutcm.edu.cn)

#1 "Diabetes Mellitus"[Mesh] 491873

#2 (diabetes) OR (DM) 948311

#3 #1 OR #2 948311

#4 "Diabetes Mellitus, Type 2"[Mesh] 163616

#5 (((((((((((((((((((((((((((((("Diabetes Mellitus, Noninsulin-Dependent") OR ("Diabetes Mellitus, Ketosis-Resistant")) OR ("Diabetes Mellitus, Ketosis Resistant")) OR ("Ketosis-Resistant Diabetes Mellitus")) OR ("Diabetes Mellitus, Non Insulin Dependent")) OR ("Diabetes Mellitus, Non-Insulin-Dependent")) OR ("Non-Insulin-Dependent Diabetes Mellitus")) OR ("Diabetes Mellitus, Stable")) OR ("Stable Diabetes Mellitus")) OR ("Diabetes Mellitus, Type II")) OR (NIDDM)) OR ("Diabetes Mellitus, Noninsulin Dependent")) OR ("Diabetes Mellitus, Maturity-Onset")) OR ("Diabetes Mellitus, Maturity Onset")) OR ("Maturity-Onset Diabetes Mellitus")) OR ("Maturity Onset Diabetes Mellitus")) OR (MODY)) OR ("Diabetes Mellitus, Slow-Onset")) OR ("Diabetes Mellitus, Slow Onset")) OR ("Slow-Onset Diabetes Mellitus")) OR ("Type 2 Diabetes Mellitus")) OR ("Noninsulin-Dependent Diabetes Mellitus")) OR ("Noninsulin Dependent Diabetes Mellitus")) OR ("Maturity-Onset Diabetes")) OR ("Diabetes, Maturity-Onset")) OR ("Maturity Onset Diabetes")) OR ("Type 2 Diabetes")) OR ("Diabetes, Type 2")) OR ("Diabetes Mellitus, Adult-Onset")) OR ("Adult-Onset Diabetes Mellitus")) OR ("Diabetes Mellitus, Adult Onset") 225318

#6 #4 OR #5 225318

#7 "Diabetes Mellitus, Type 1"[Mesh] 84523

#8 (((((((((((((((((((((((((("Diabetes Mellitus, Insulin-Dependent") OR ("Diabetes Mellitus, Insulin Dependent")) OR ("Insulin-Dependent Diabetes Mellitus")) OR ("Diabetes Mellitus, Juvenile-Onset")) OR ("Diabetes Mellitus, Juvenile Onset")) OR ("Juvenile-Onset Diabetes Mellitus")) OR (IDDM)) OR ("Juvenile-Onset Diabetes")) OR ("Diabetes, Juvenile-Onset")) OR ("Juvenile Onset Diabetes")) OR ("Diabetes Mellitus, Sudden-Onset")) OR ("Diabetes Mellitus, Sudden Onset")) OR ("Sudden-Onset Diabetes Mellitus")) OR ("Type 1 Diabetes Mellitus")) OR ("Diabetes Mellitus, Insulin-Dependent, 1")) OR ("Insulin-Dependent Diabetes Mellitus 1")) OR ("Insulin Dependent Diabetes Mellitus 1")) OR ("Type 1 Diabetes")) OR ("Diabetes, Type 1")) OR ("Diabetes Mellitus, Type I")) OR ("Diabetes Mellitus, Brittle")) OR ("Brittle Diabetes Mellitus")) OR ("Diabetes Mellitus, Ketosis-Prone")) OR ("Diabetes Mellitus, Ketosis Prone")) OR ("Ketosis-Prone Diabetes Mellitus")) OR ("Diabetes, Autoimmune")) OR ("Autoimmune Diabetes") 108637

#9 #7 OR #8 108637

#10 #3 OR #6 OR #9 950590

#11 (((((((((((("Potentilla discolor Bunge") OR ("Potentilla discolor")) OR ("Herba Potentillae Discoloris")) OR OR (Potentilla)) OR ("Potentilla discolor Bge")) OR ("aqueous extract of Potentilla discolor ")) OR (" Potentilla discolor decoction ")) OR ("flavonoids from Potentilla discolor ")) OR ("Potentilla discolor Aqueous Extract")) OR ("aqueous extract of Potentilla discolor Bunge")) OR ("Potentillae Discoloris Herb")) OR ("Crude Triterpenes Extract from Fanbaricao")) OR ("Crude Triterpenes Extract from Potentilla discolor") 1126

#12 #10 AND #11 74
