# Supplementary material for: Effects of potentilla discolor bunge extracts on oxidative stress and glycolipid metabolism in animal models of diabetes: a systematic review and meta-analysis
Source: Front Pharmacol. 2023 Oct 2;14:1218757. doi: 10.3389/fphar.2023.1218757 (PMC10577192; doi:10.3389/fphar.2023.1218757)
Supplement: Supplementary file 3 [file Table5.DOCX]

***Supplementary Material 5-******Results of trim-and-fill method***

**Effects of Potentilla discolor Bunge extracts on oxidative stress and glycolipid metabolism in diabetic animal models: A systematic review and meta-analysis**

Yunjiao Yang, Wen Deng, Yue Wu, Changyan Zi, Qiu Chen^,^*

***Corresponding author:** Qiu Chen

E-mail: [chenqiu1005@cdutcm.edu.cn](mailto:chenqiu1005@cdutcm.edu.cn)


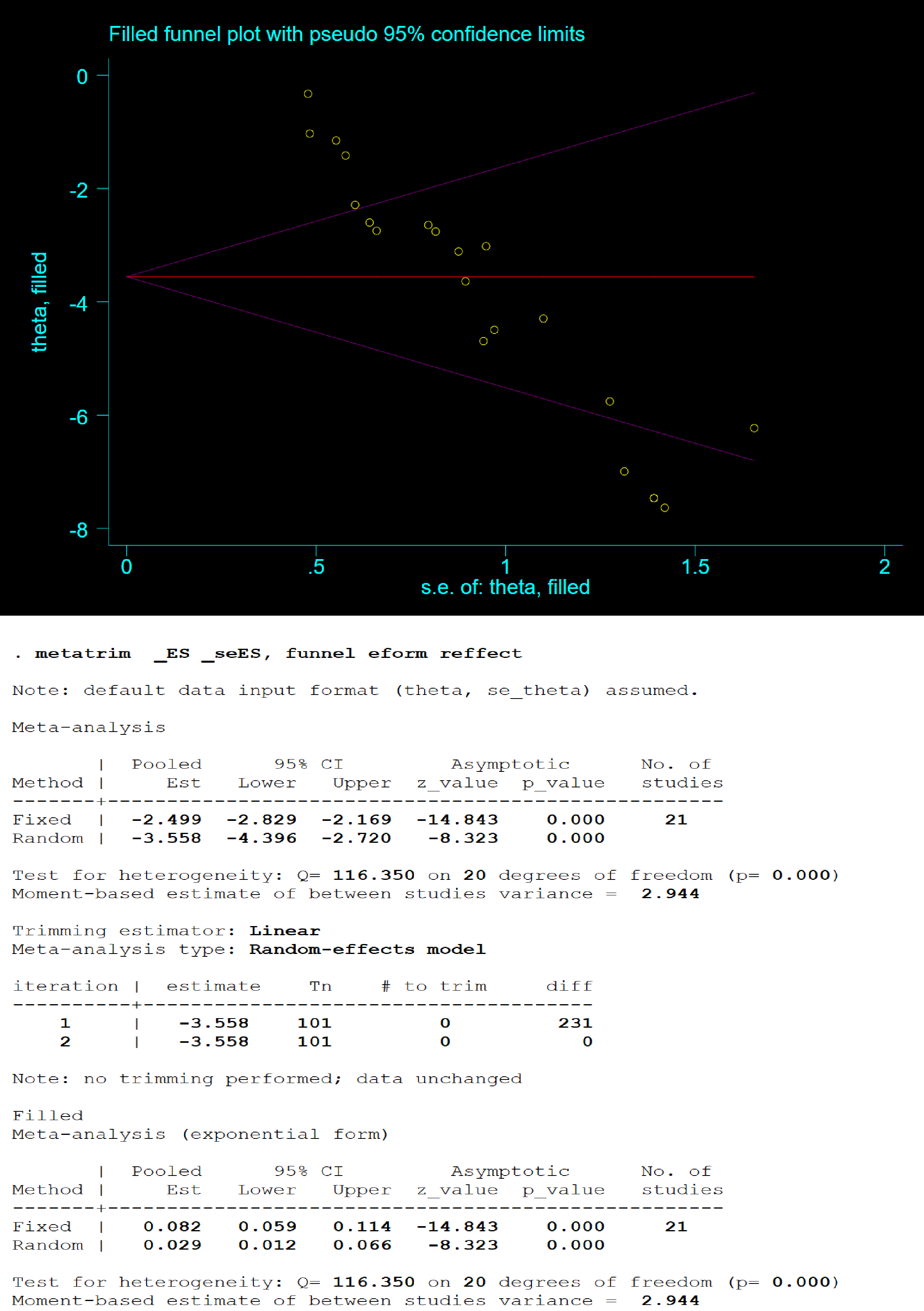


**Figure 1.** Result of trim-and-fill method for the effect of PDB extracts on FBG


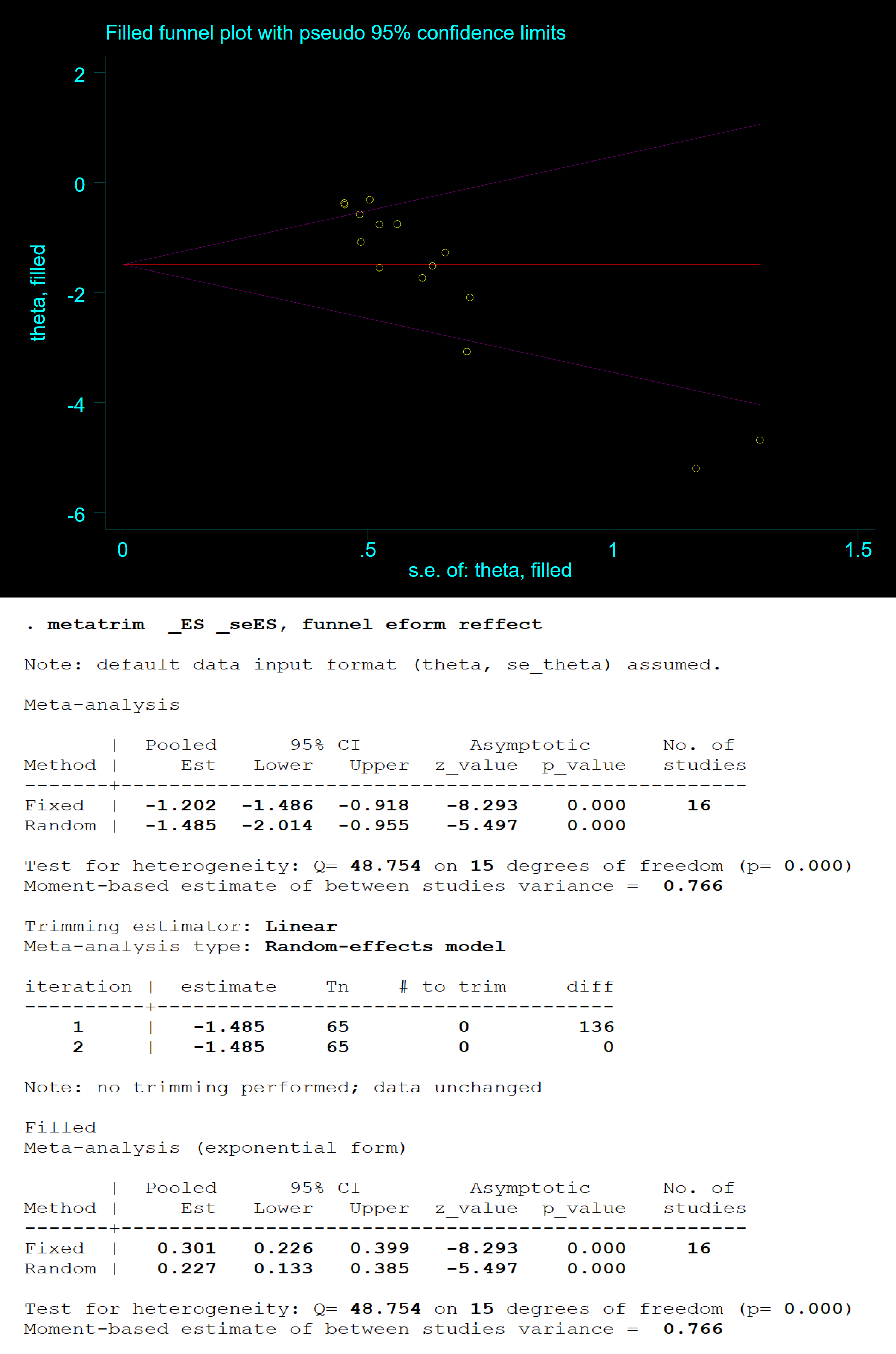


**Figure 2.** Result of trim-and-fill method for the effect of PDB extracts on TG


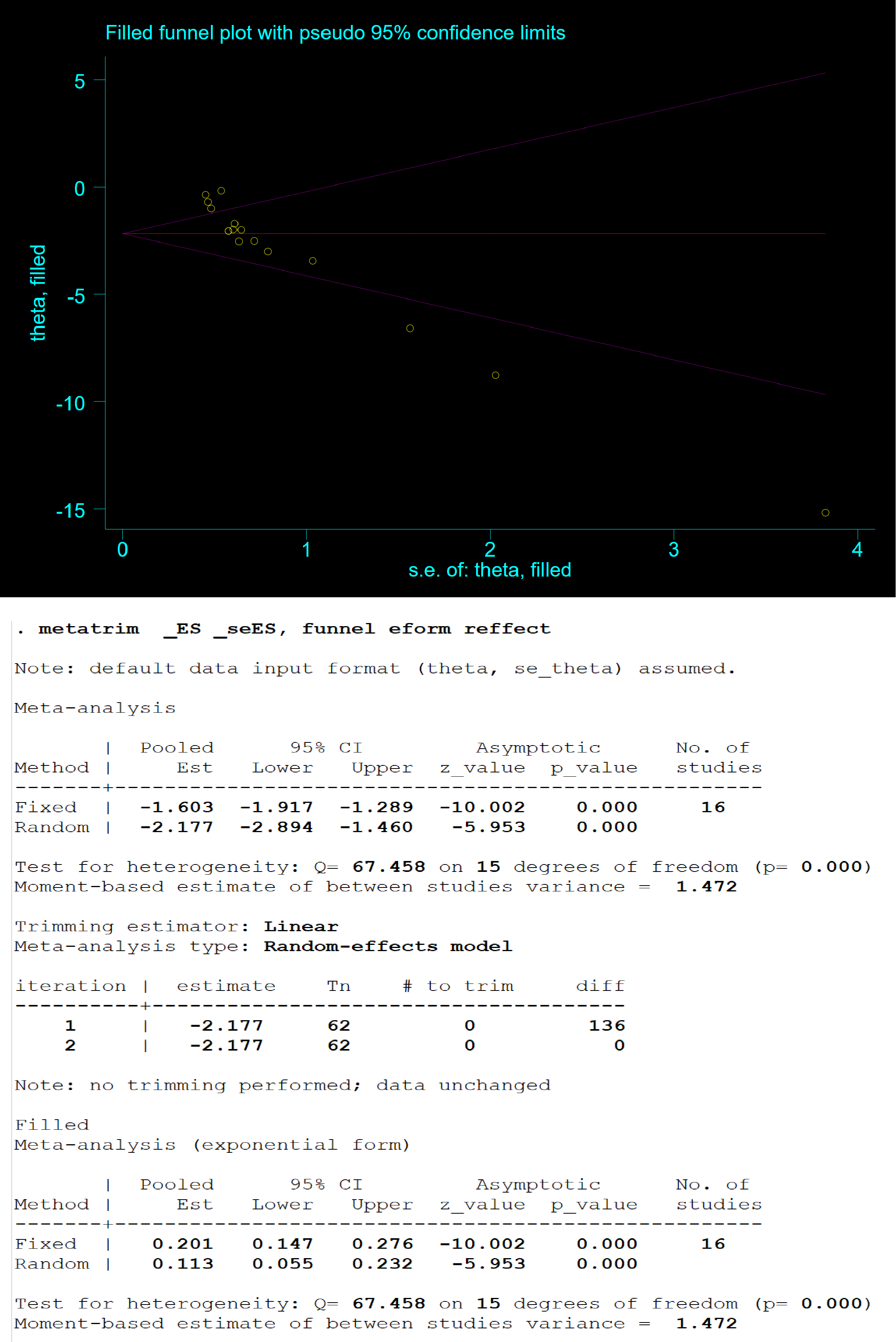


**Figure 3.** Result of trim-and-fill method for the effect of PDB extracts on TC


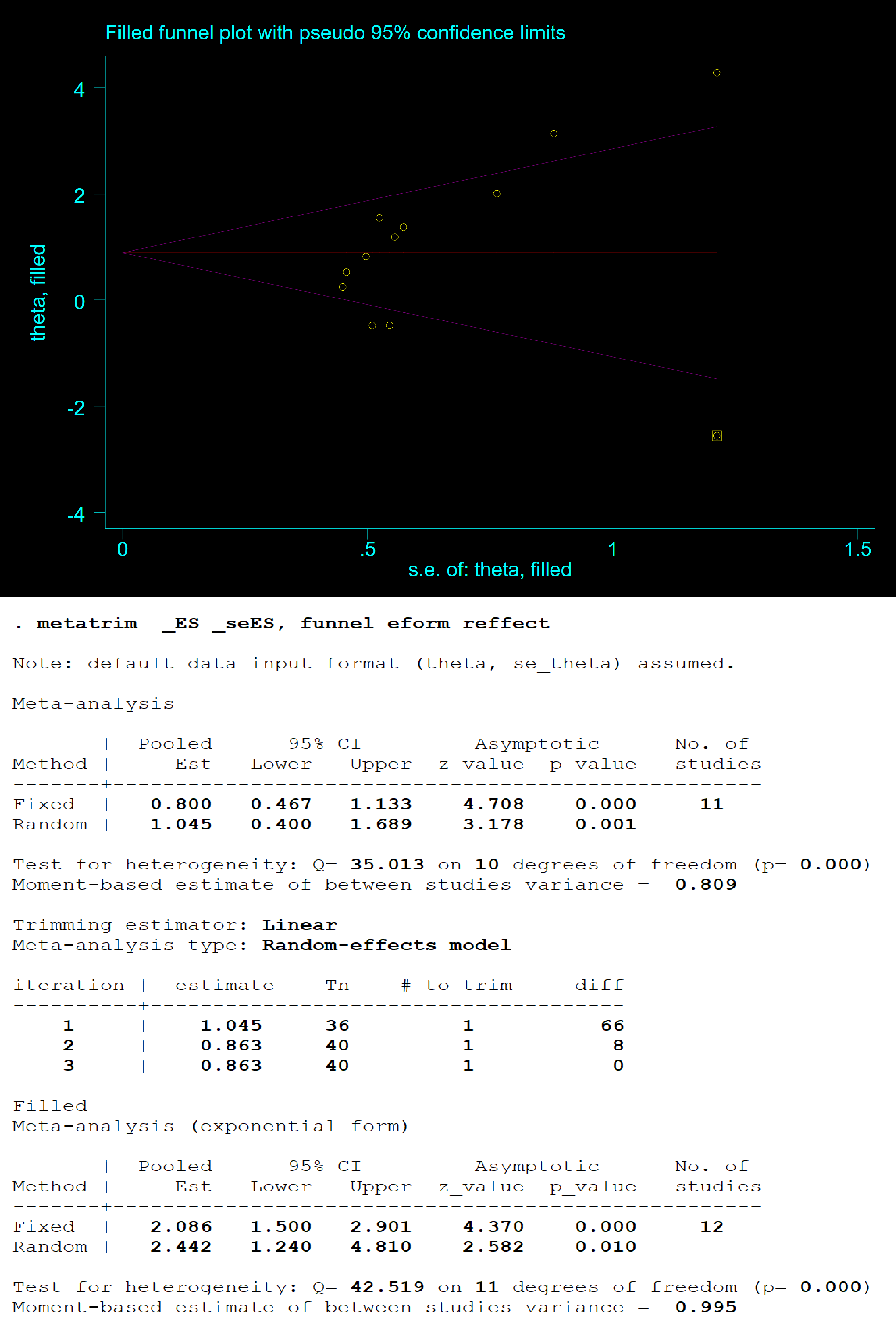


**Figure 4.** Result of trim-and-fill method for the effect of PDB extracts on HDL-C


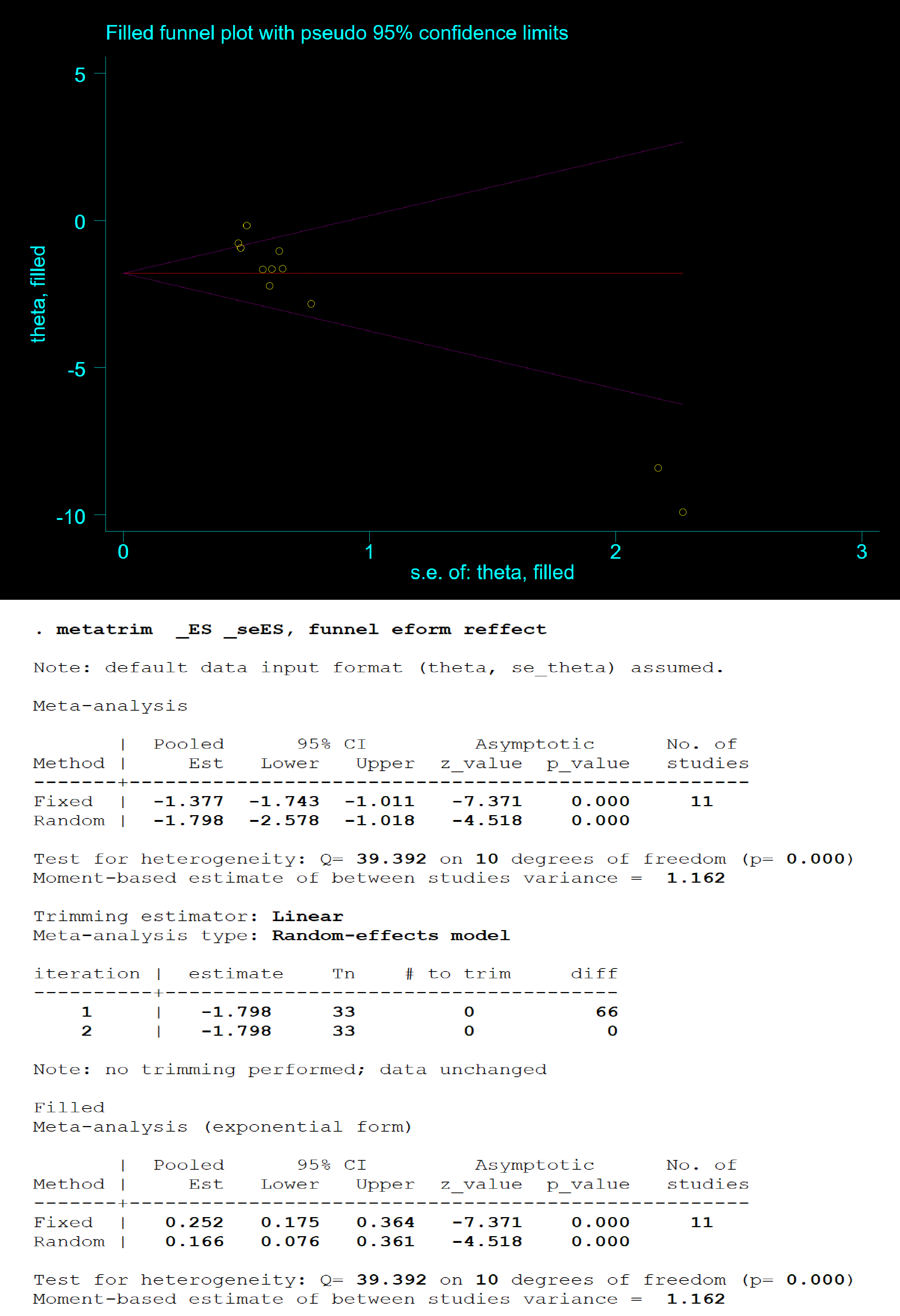


**Figure 5.** Result of trim-and-fill method for the effect of PDB extracts on LDL-C


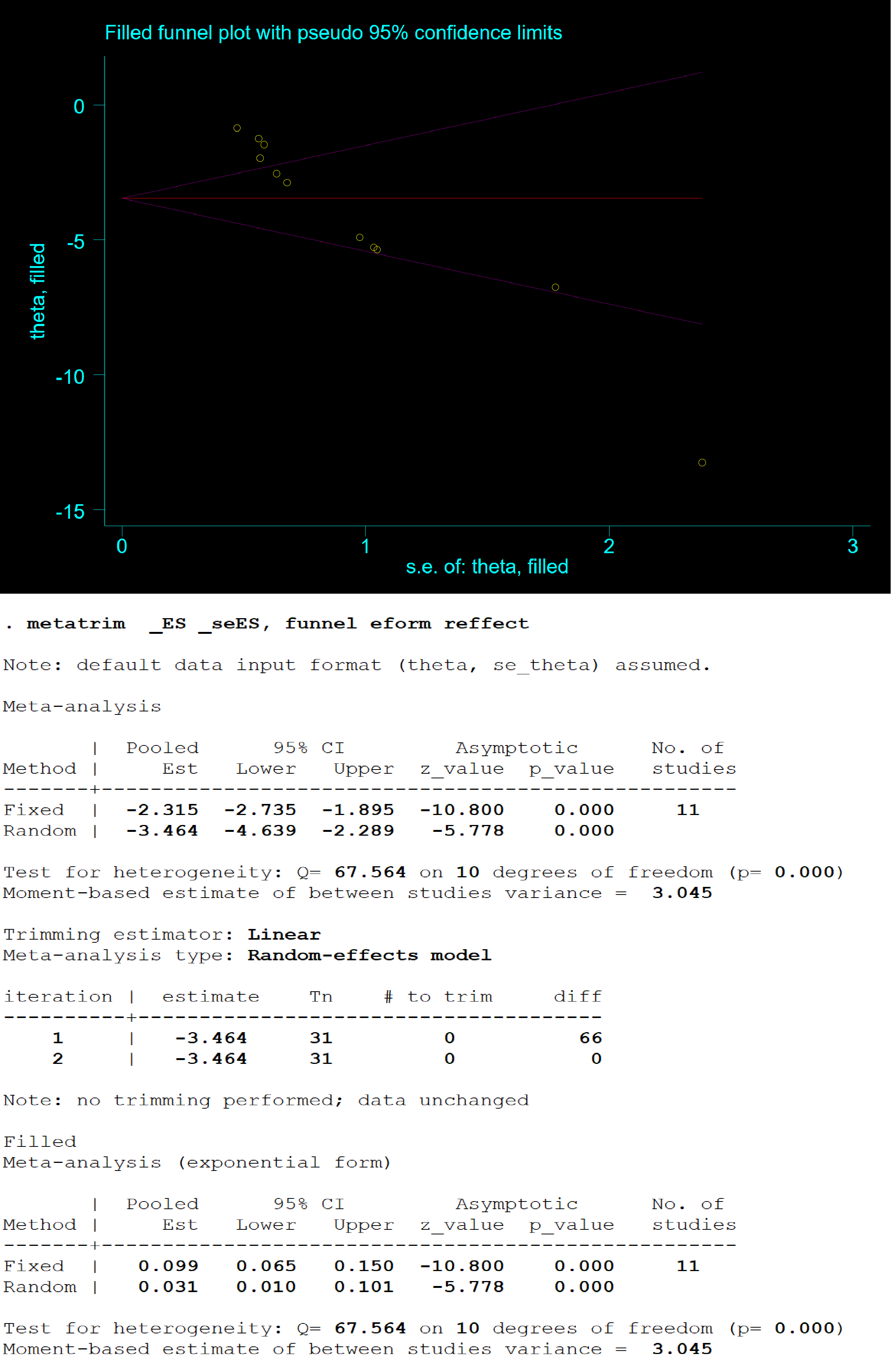


**Figure 6.** Result of trim-and-fill method for the effect of PDB extracts on MDA


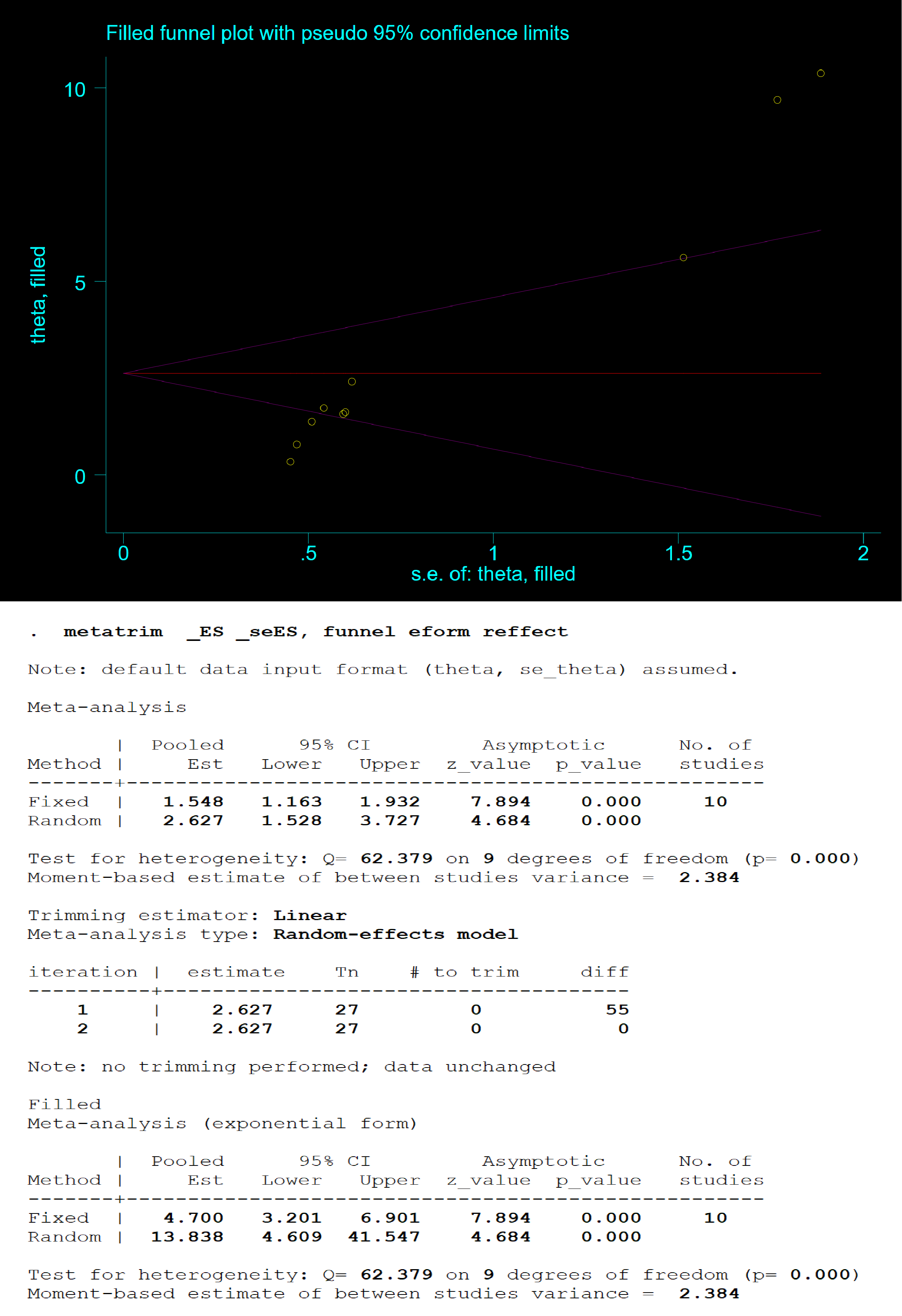


**Figure 7.** Result of trim-and-fill method for the effect of PDB extracts on SOD
