# Supplementary material for: Effects of potentilla discolor bunge extracts on oxidative stress and glycolipid metabolism in animal models of diabetes: a systematic review and meta-analysis
Source: Front Pharmacol. 2023 Oct 2;14:1218757. doi: 10.3389/fphar.2023.1218757 (PMC10577192; doi:10.3389/fphar.2023.1218757)
Supplement: Supplementary file 4 [file Table3.DOCX]

***Supplementary Material 3-Figures of sensitivity analyses***

**Effects of Potentilla discolor Bunge extracts on oxidative stress and glycolipid metabolism in diabetic animal models: A systematic review and meta-analysis**

**Yunjiao Yang, Wen Deng, Yue Wu,** **Changyan Zi, Qiu Chen^,^***

***Corresponding author:** Qiu Chen

E-mail: [chenqiu1005@cdutcm.edu.cn](mailto:chenqiu1005@cdutcm.edu.cn)


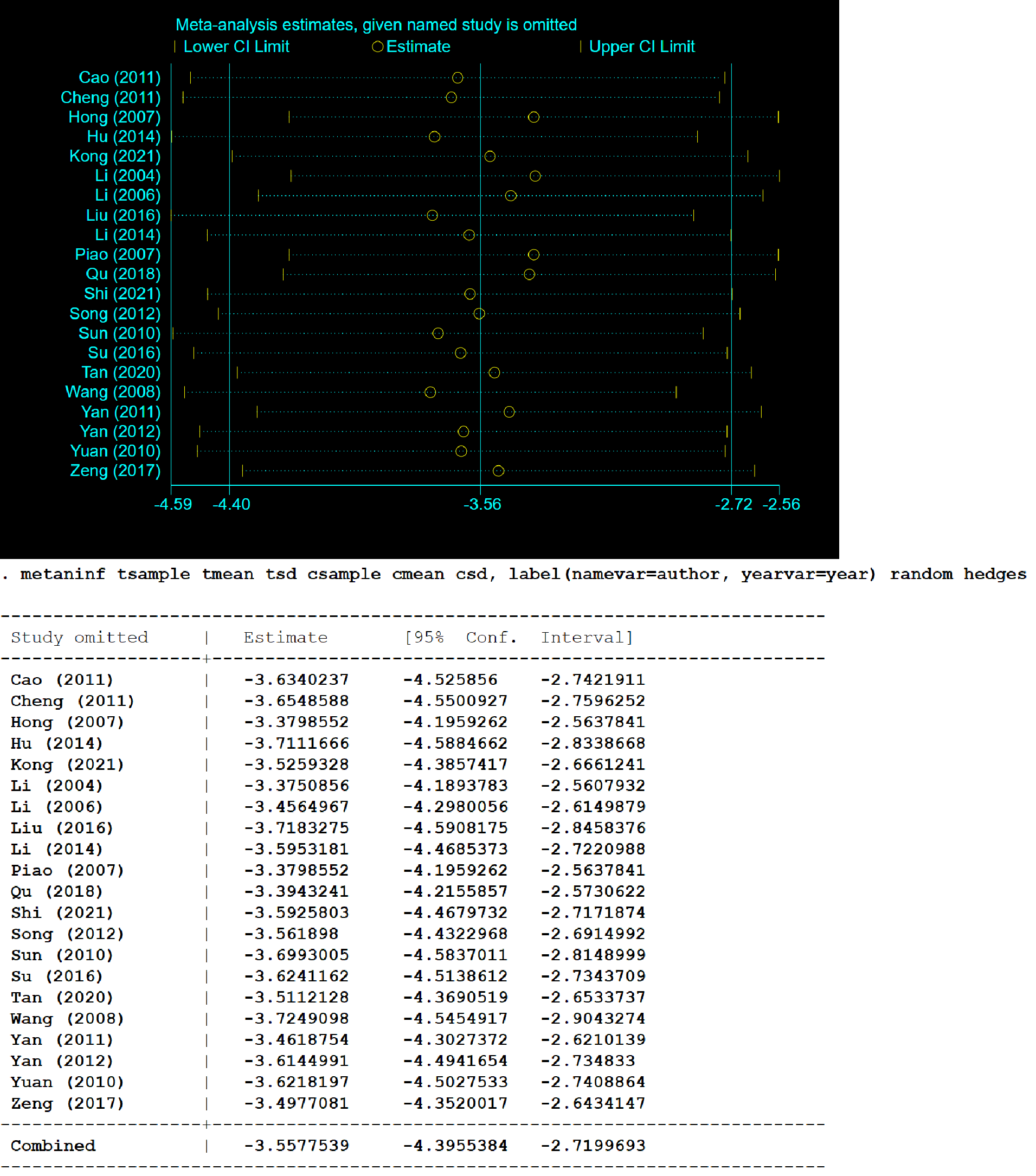


**Figure 1. Sensitivity analysis of the effect of** **PDB extracts on FBG in diabetic animals**


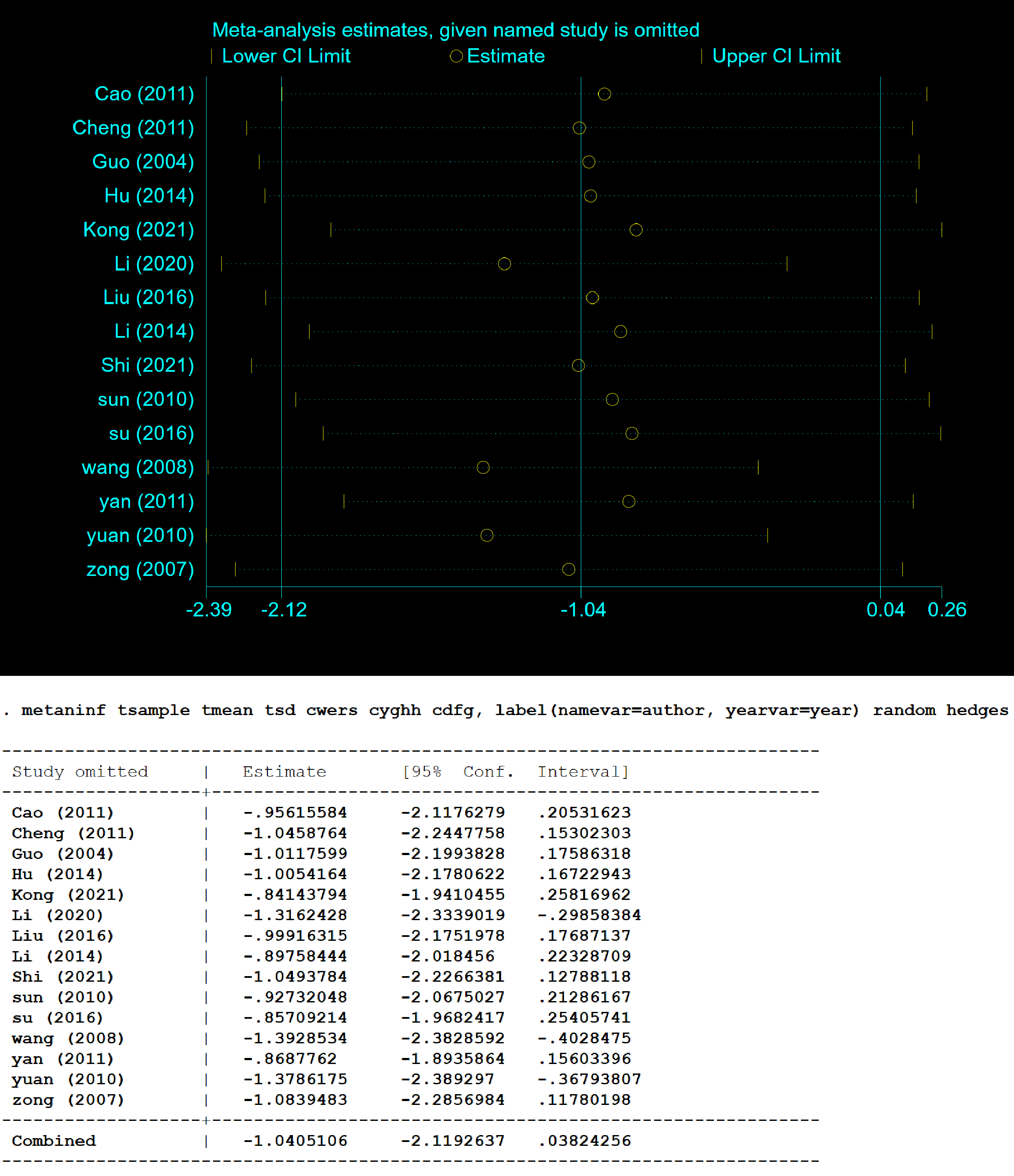


**Figure 2. Sensitivity analysis of the effect of PDB extracts on FINS in diabetic animals**


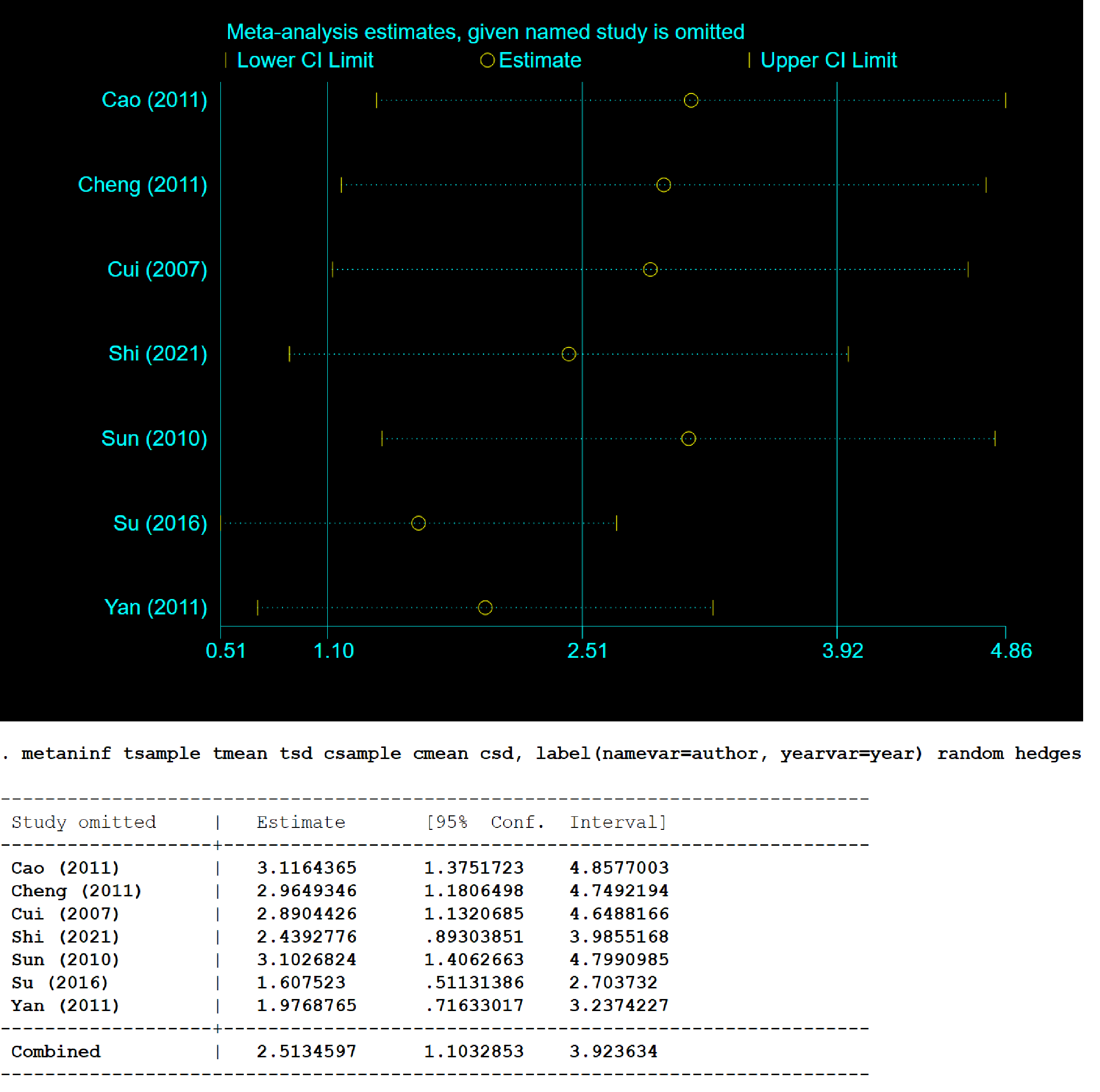


**Figure 3. Sensitivity analysis of the effect of PDB extracts on ISI in diabetic animals**


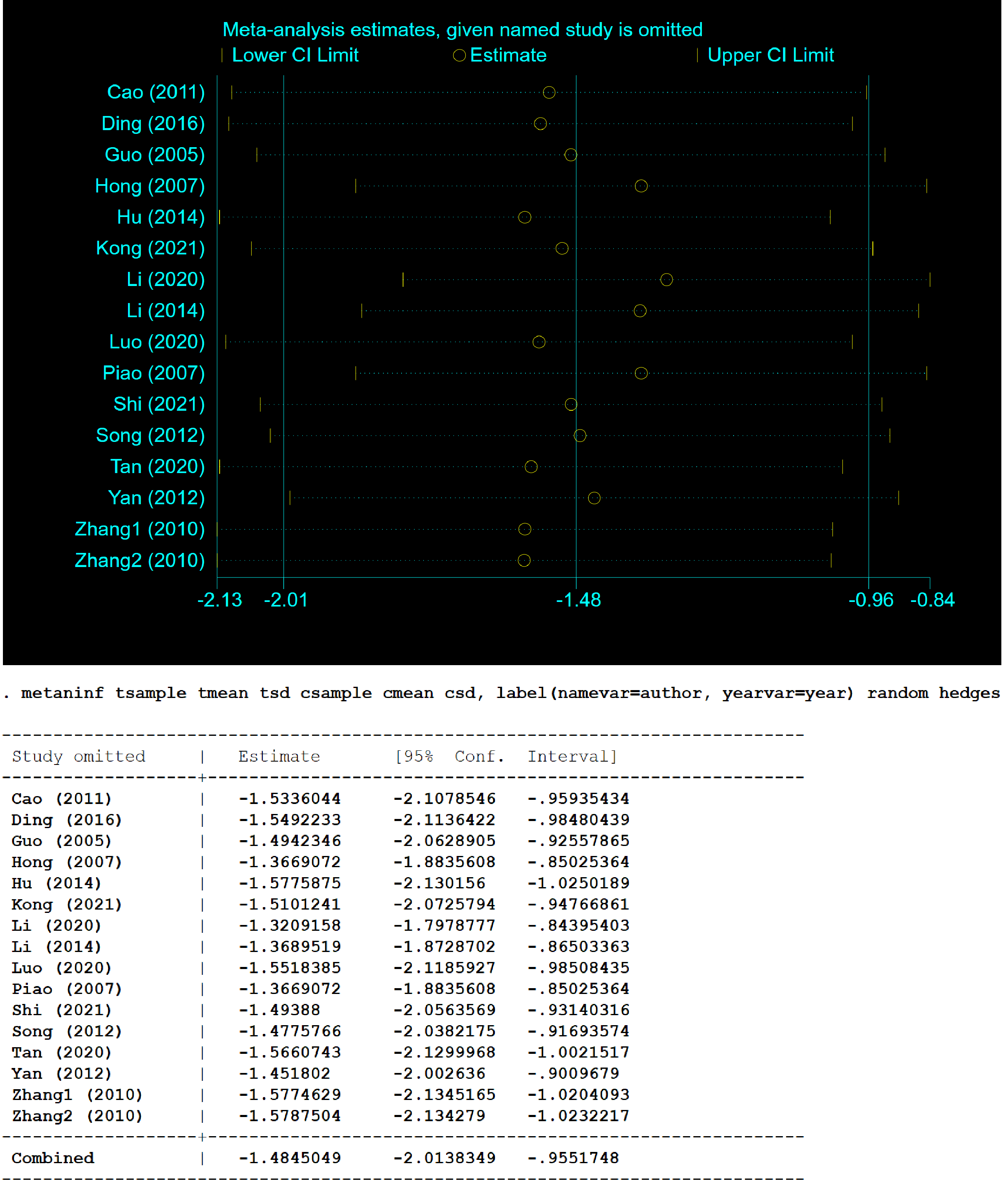


**Figure 4. Sensitivity analysis of the effect of PDB extracts on TG in diabetic animals**


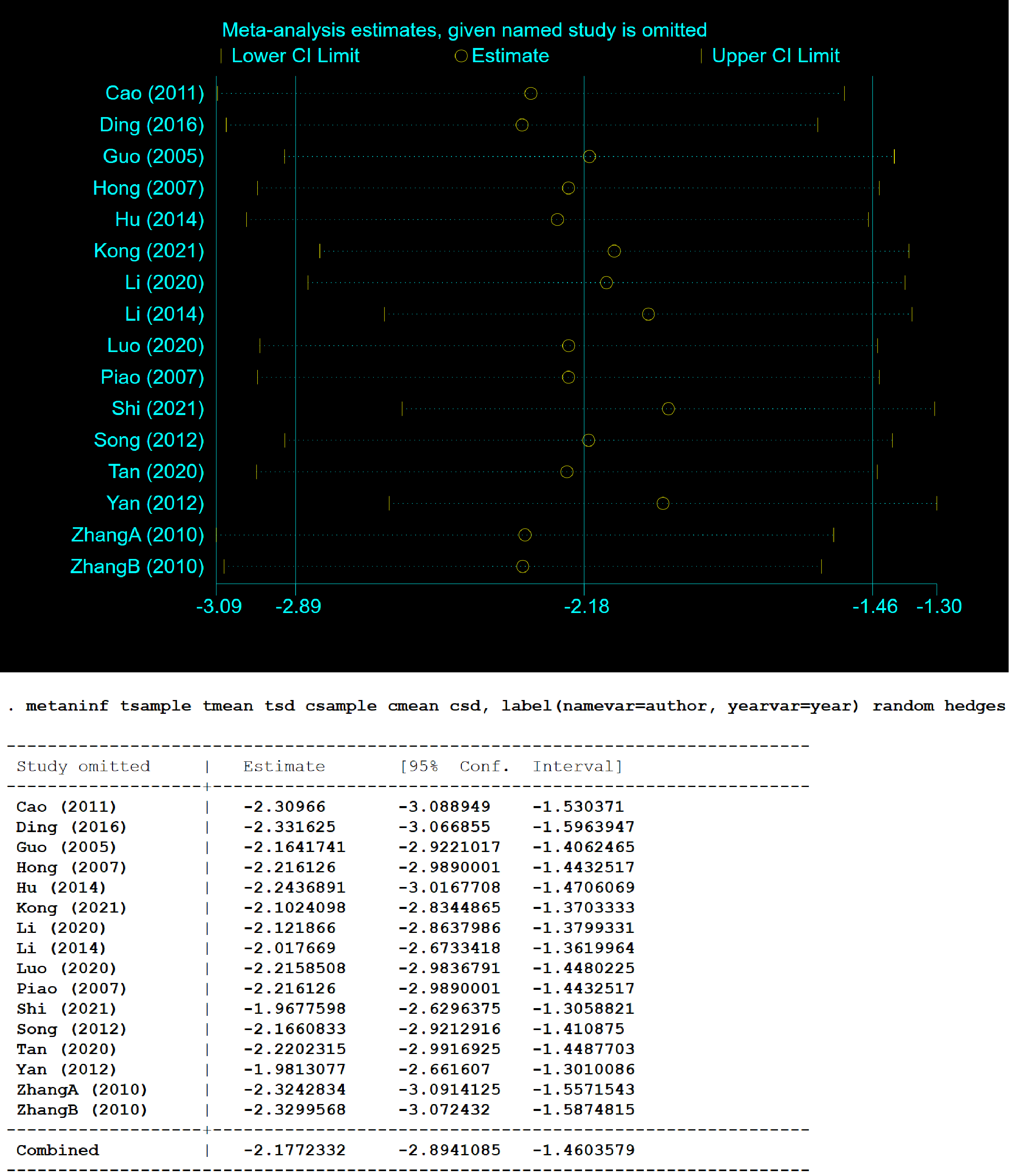


**Figure 5. Sensitivity analysis of the effect of PDB extracts on TC in diabetic animals**


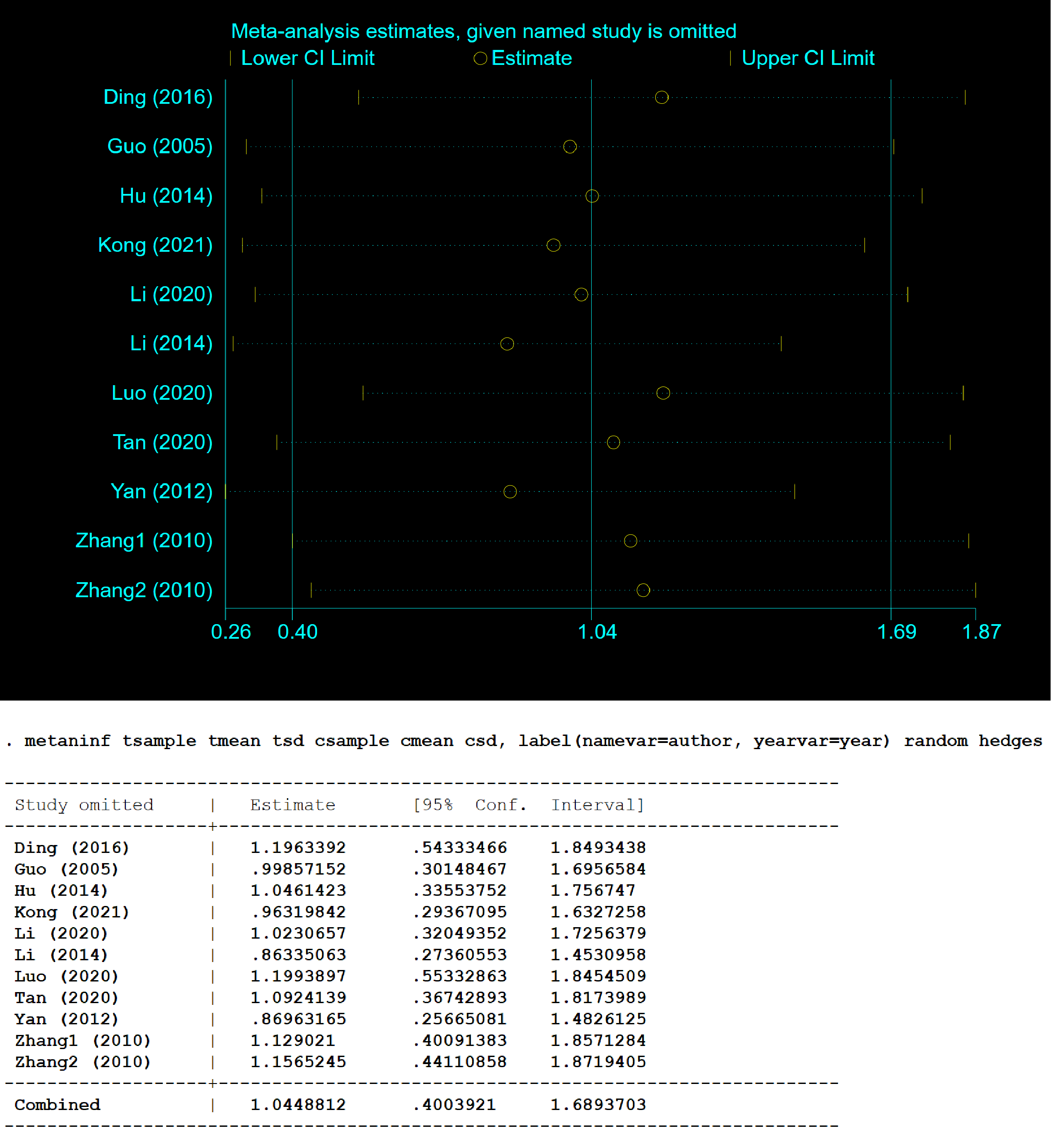


**Figure 6. Sensitivity analysis of the effect of PDB extracts on HDL-C in diabetic animals**


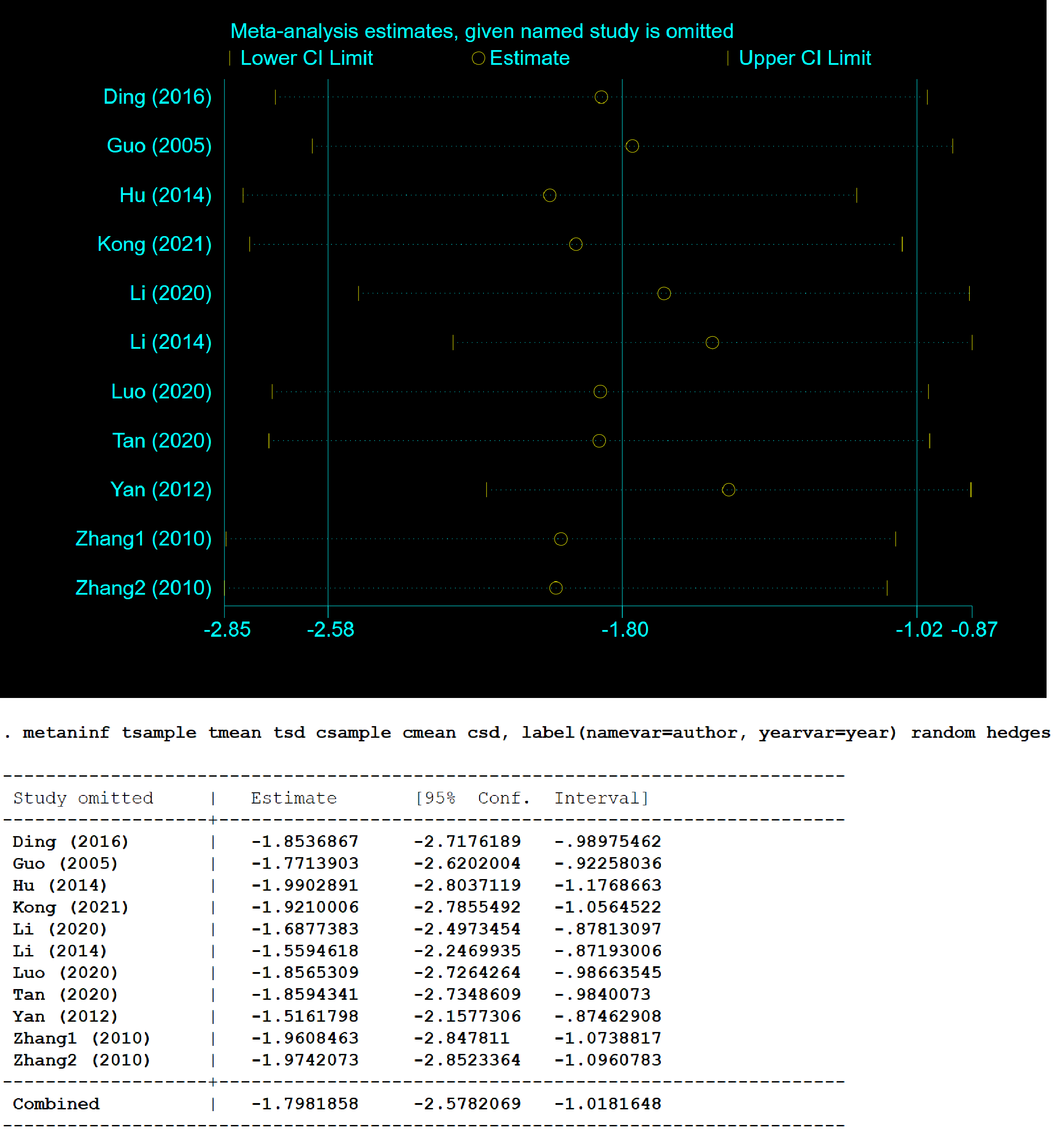


**Figure 7. Sensitivity analysis of the effect of PDB extracts on LDL-C in diabetic animals**


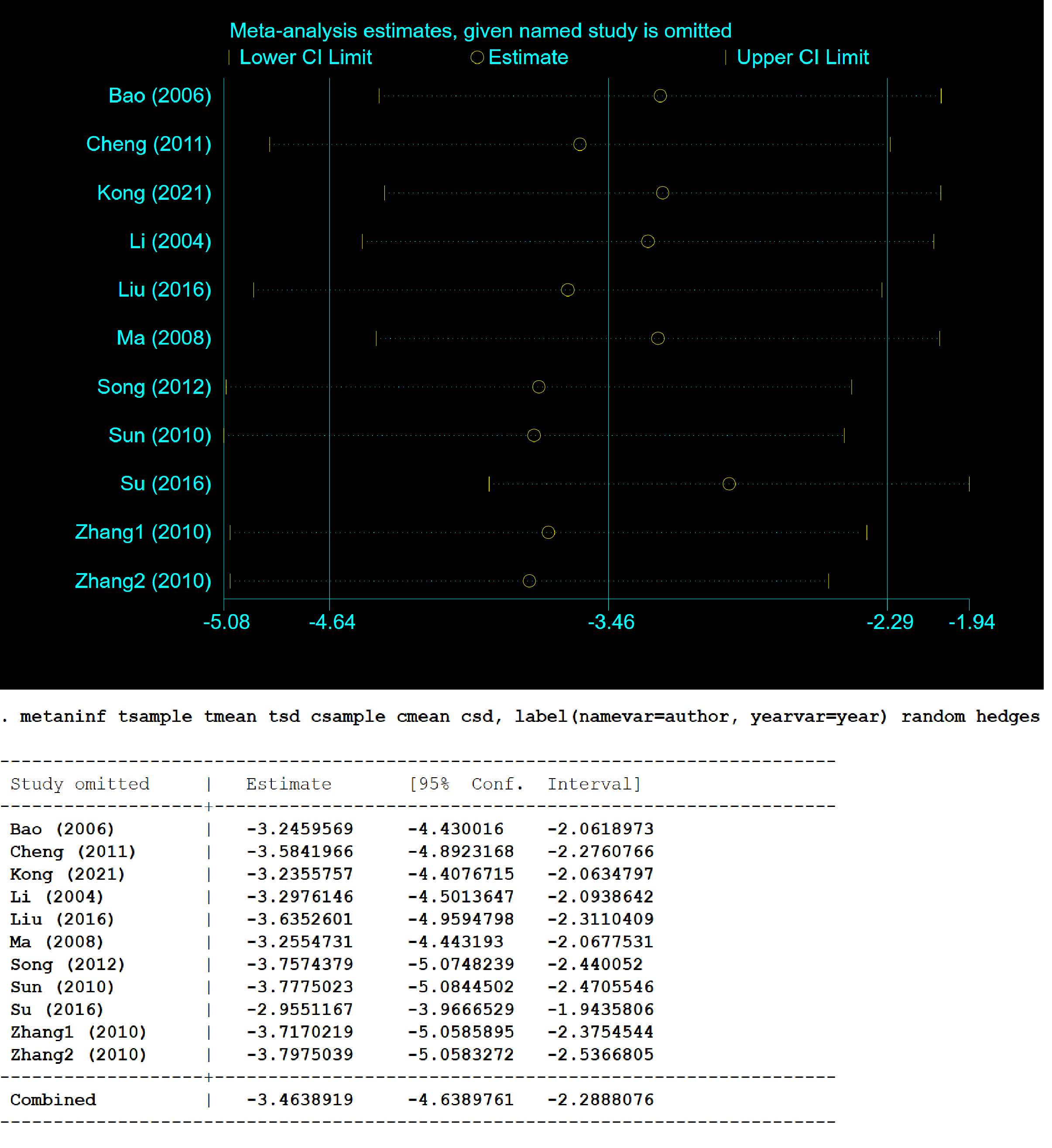


**Figure 8. Sensitivity analysis of the effect of PDB extracts on MDA in diabetic animals**


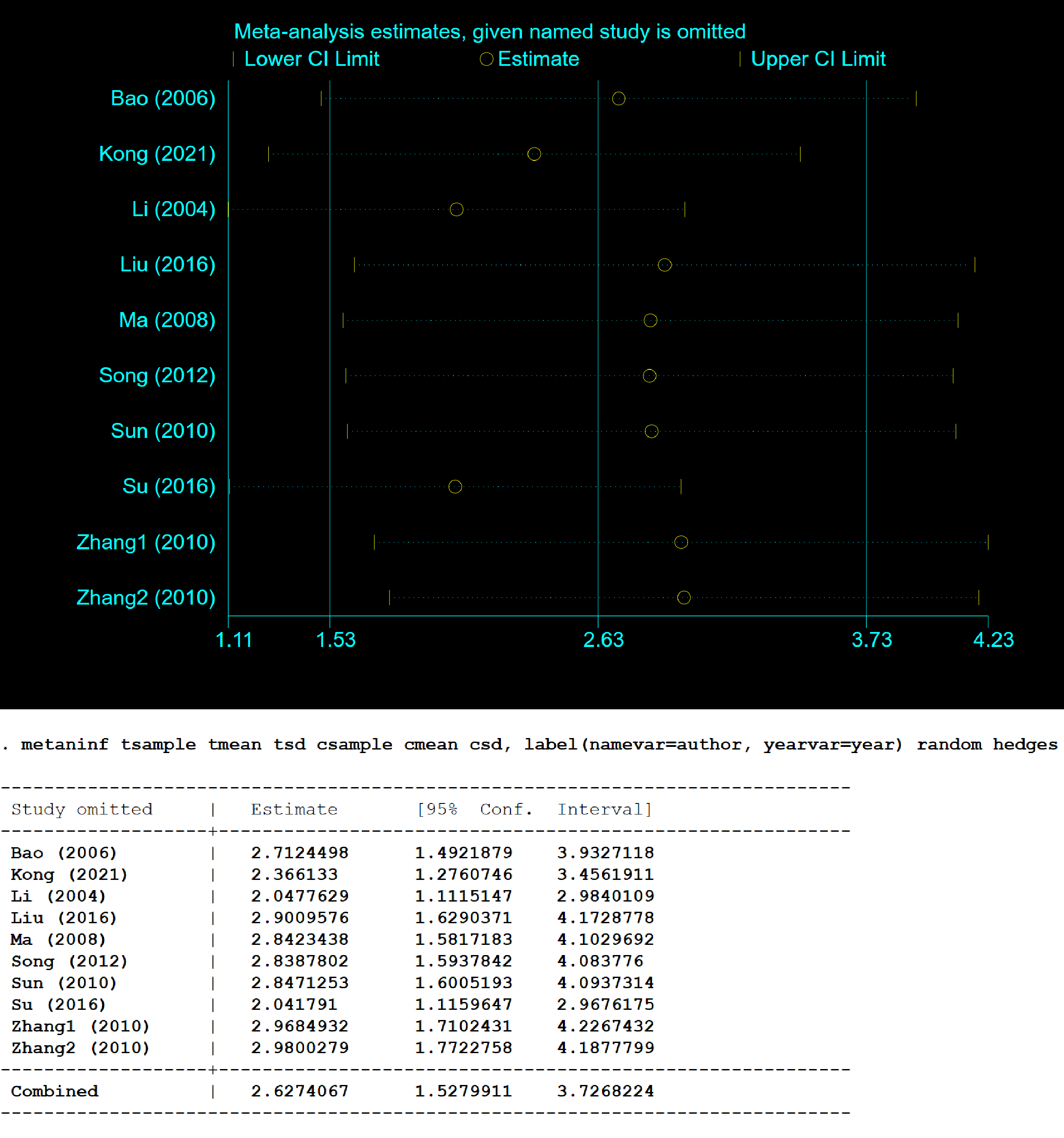


**Figure 9. Sensitivity analysis of the effect of PDB extracts on SOD in diabetic animals**


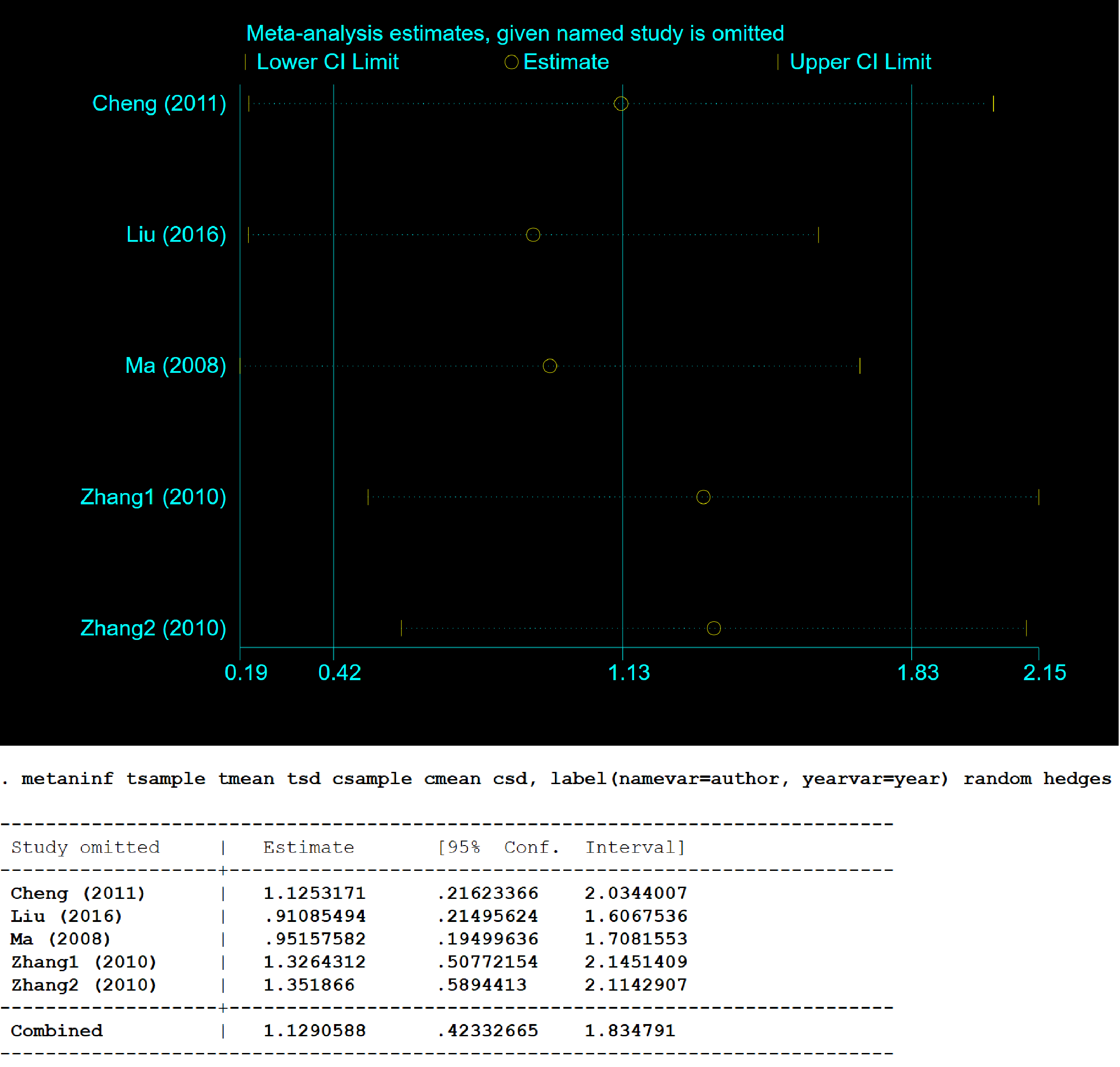


**Figure 10. Sensitivity analysis of the effect of PDB extracts on GSH-px in diabetic animals**


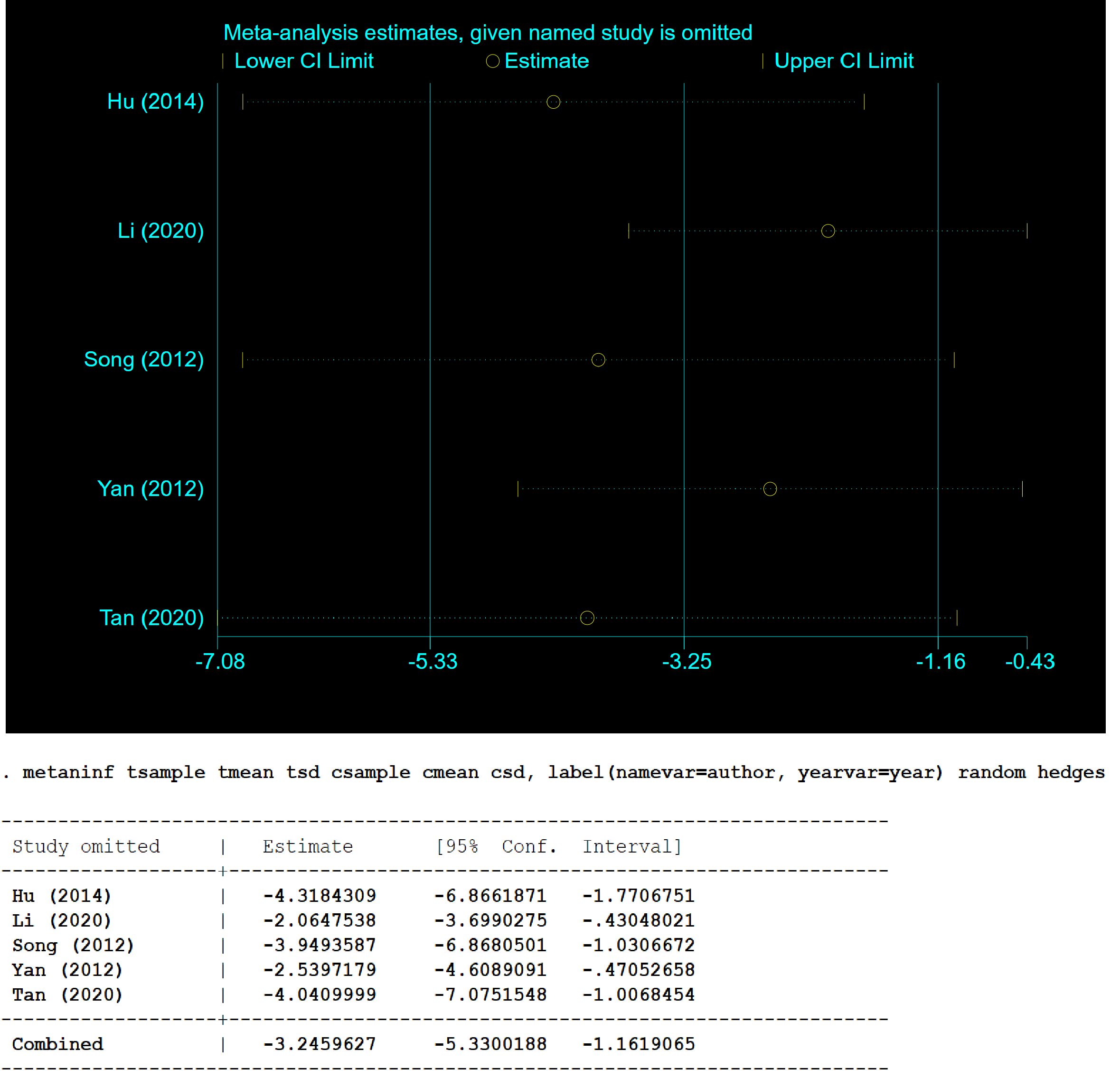


**Figure 11. Sensitivity analysis of the effect of PDB extracts on FFA in diabetic animals**


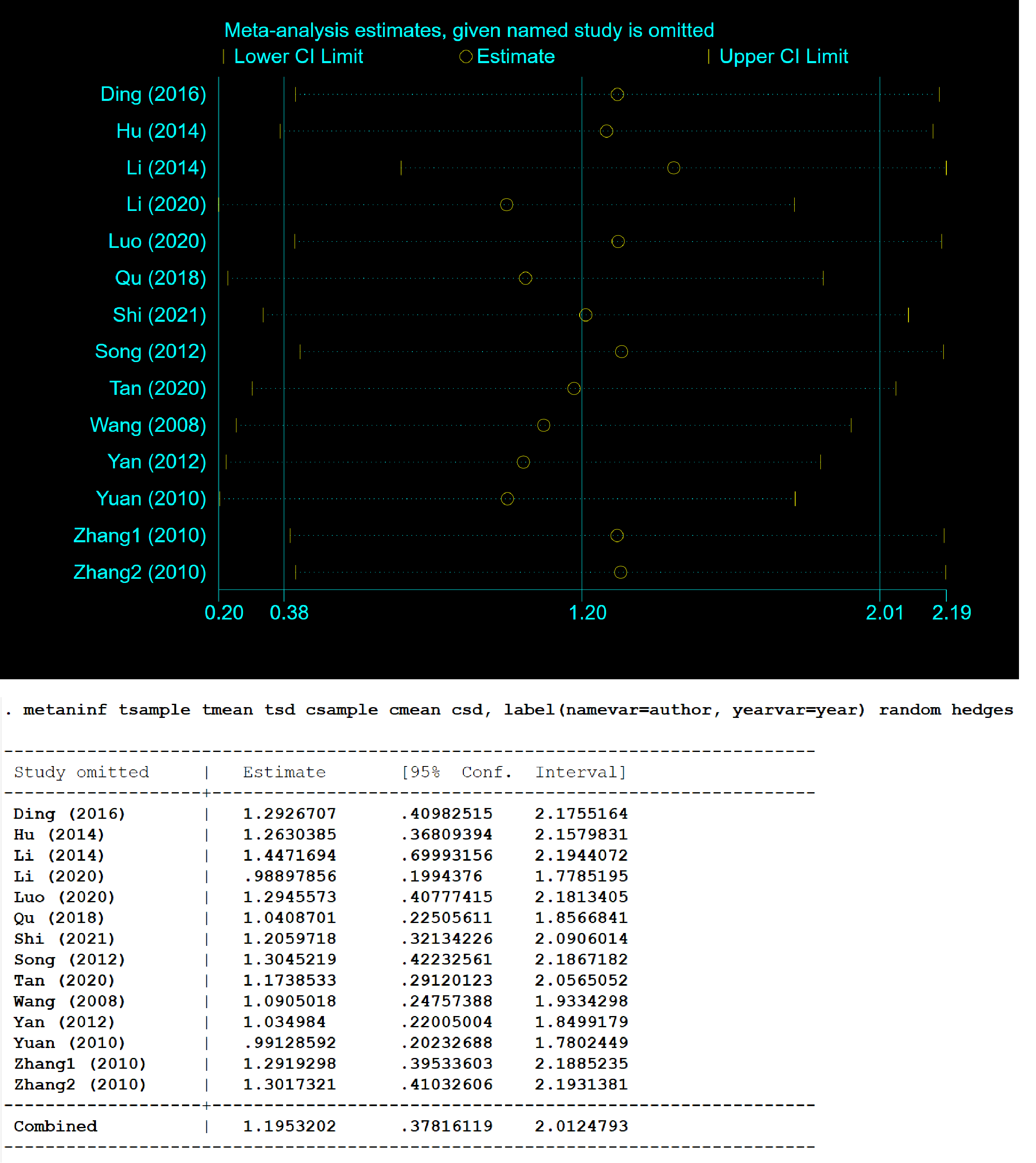


**Figure 12. Sensitivity analysis of the effect of PDB extracts on body weight in diabetic animals**
