# Supplementary material for: Effects of potentilla discolor bunge extracts on oxidative stress and glycolipid metabolism in animal models of diabetes: a systematic review and meta-analysis
Source: Front Pharmacol. 2023 Oct 2;14:1218757. doi: 10.3389/fphar.2023.1218757 (PMC10577192; doi:10.3389/fphar.2023.1218757)
Supplement: Supplementary file 5 [file Table4.DOCX]

***Supplementary Material 3-Results of publication bias***

**Effects of Potentilla discolor Bunge extracts on oxidative stress and glycolipid metabolism in diabetic animal models: A systematic review and meta-analysis**

Yunjiao Yang, Wen Deng, Yue Wu, Changyan Zi, Qiu Chen^,^*

***Corresponding author:** Qiu Chen

E-mail: [chenqiu1005@cdutcm.edu.cn](mailto:chenqiu1005@cdutcm.edu.cn)


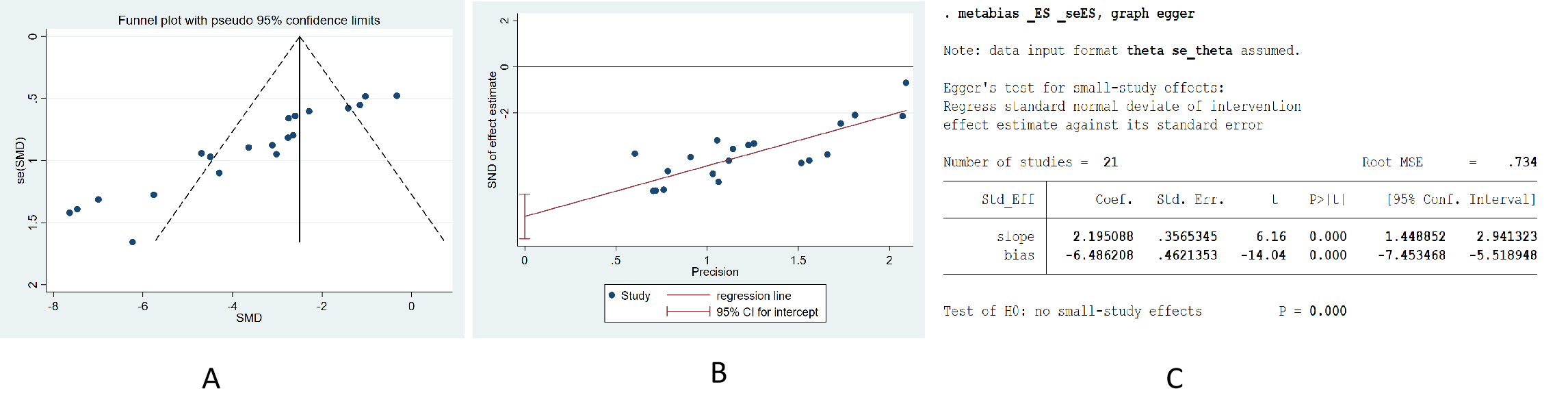


**Figure 1.** Publication bias of the effect of PDB extracts on **FBG**. (A) Funnel plot of publication bias; (B) Graph of egger^,^s test; (C) Result of egger^,^s test


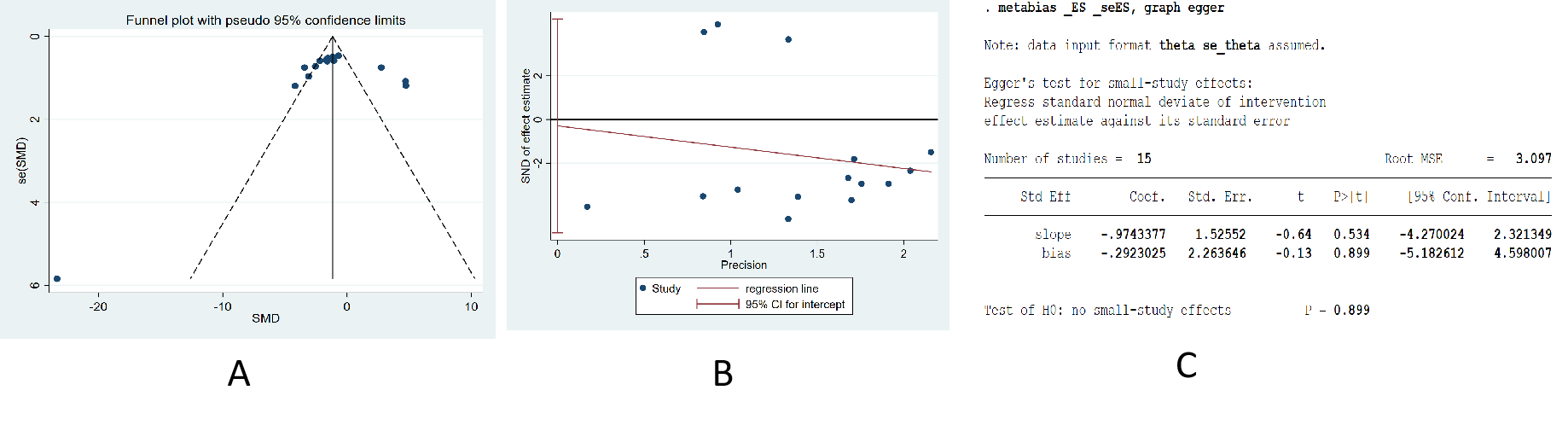
**Figure 2.** Publication bias of the effect of PDB extracts on **FINS**. (A) Funnel plot of publication bias; (B) Graph of egger^,^s test; (C) Result of egger^,^s test


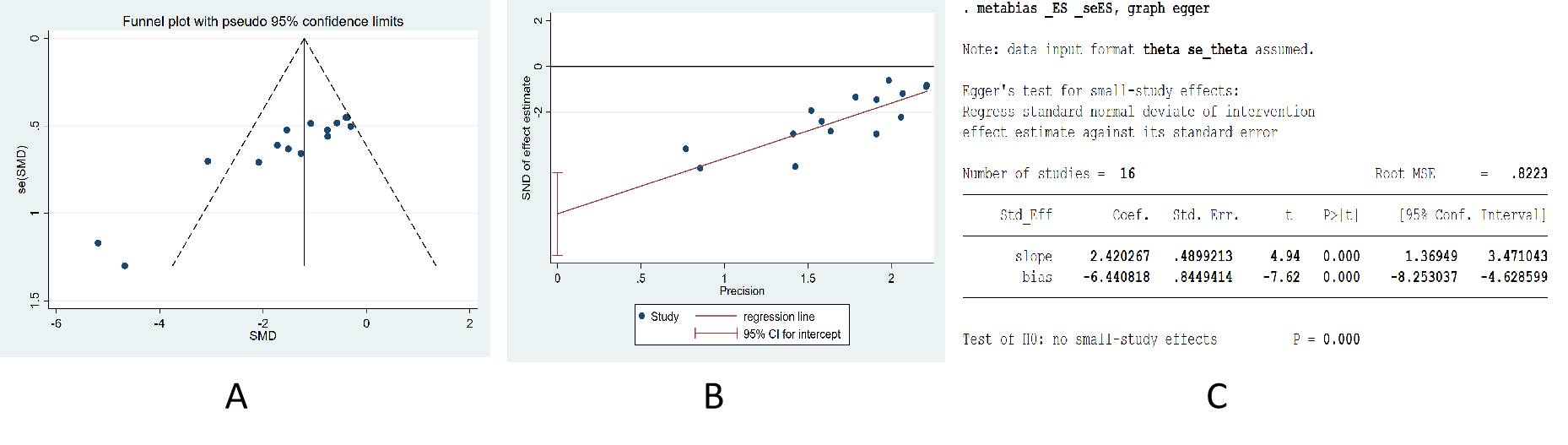
**Figure 3.** Publication bias of the effect of PDB extracts on **TG**. (A) Funnel plot of publication bias; (B) Graph of egger^,^s test; (C) Result of egger^,^s test


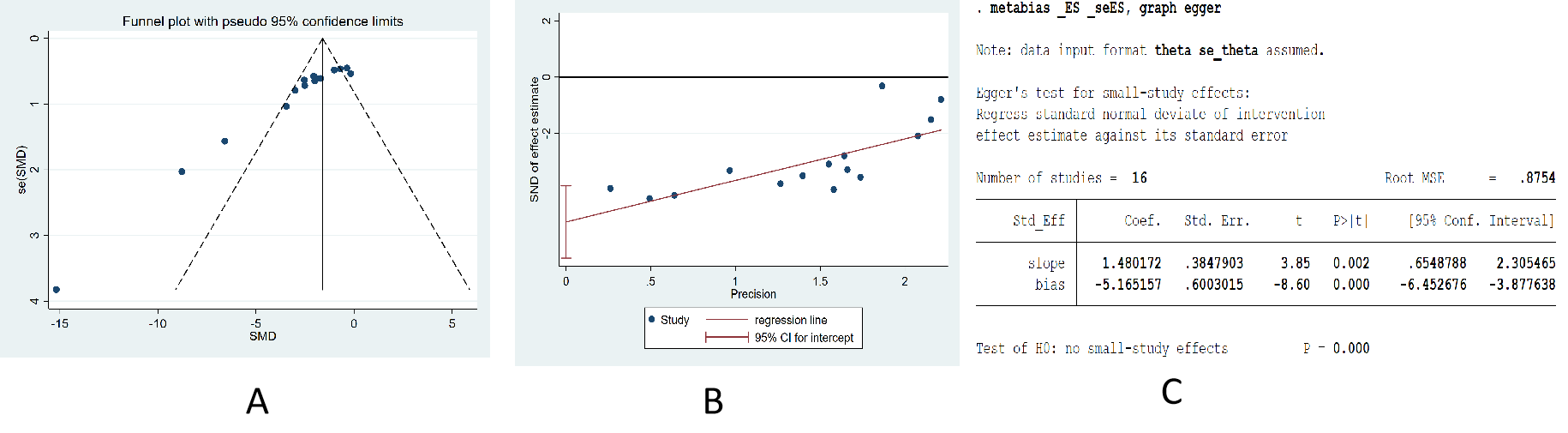


**Figure 4.** Publication bias of the effect of PDB extracts on **TC**. (A) Funnel plot of publication bias; (B) Graph of egger^,^s test; (C) Result of egger^,^s test


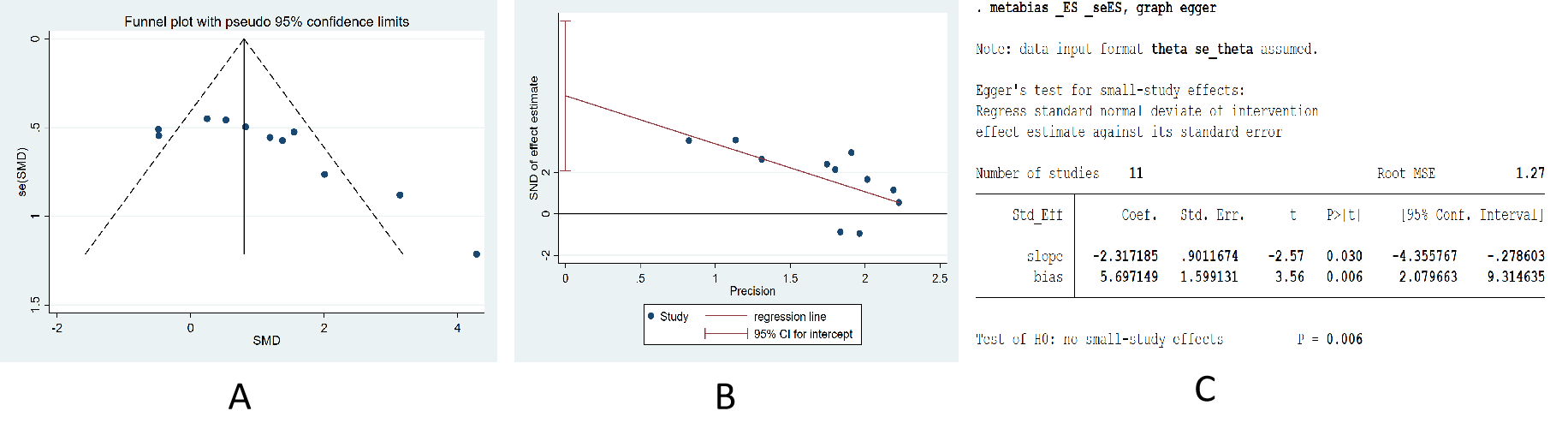


**Figure 5.** Publication bias of the effect of PDB extracts on **HDL-C**. (A) Funnel plot of publication bias; (B) Graph of egger^,^s test; (C) Result of egger^,^s test


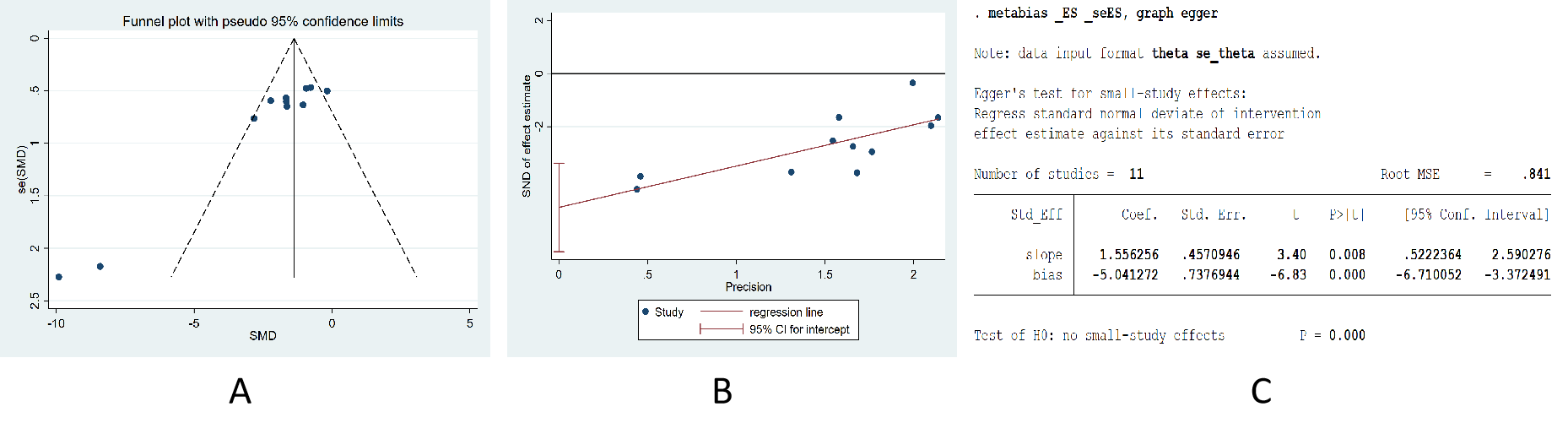


**Figure 6.** Publication bias of the effect of PDB extracts on **LDL-C**. (A) Funnel plot of publication bias; (B) Graph of egger^,^s test; (C) Result of egger^,^s test


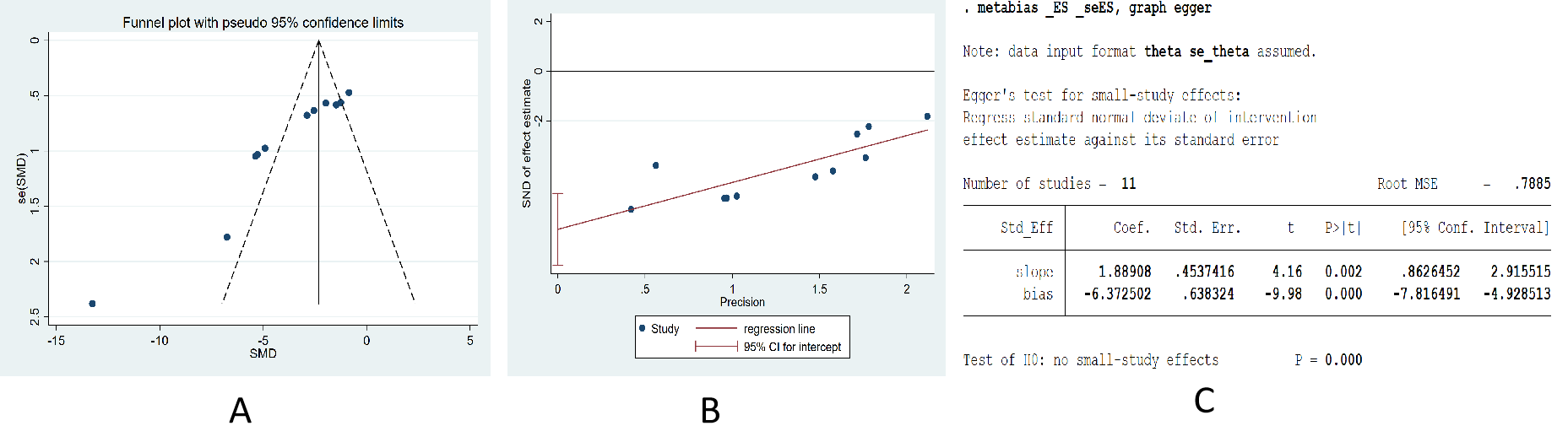


**Figure 7.** Publication bias of the effect of PDB extracts on **MDA**. (A) Funnel plot of publication bias; (B) Graph of egger^,^s test; (C) Result of egger^,^s test


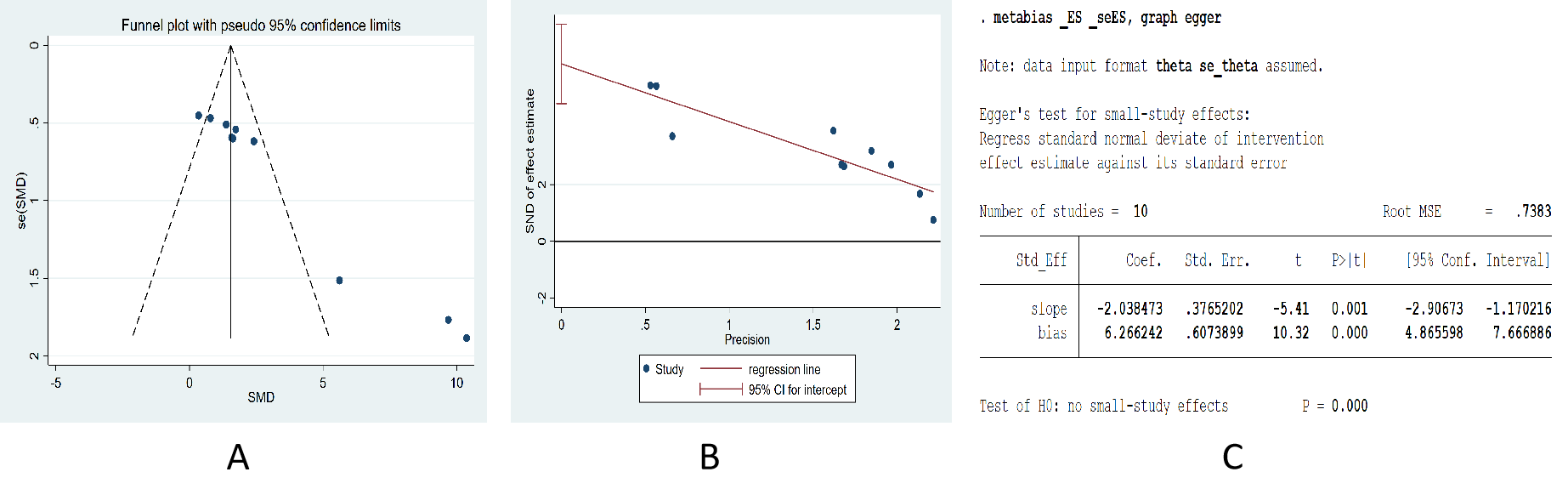


**Figure 8.** Publication bias of the effect of PDB extracts on **SOD**. (A) Funnel plot of publication bias; (B) Graph of egger^,^s test; (C) Result of egger^,^s test


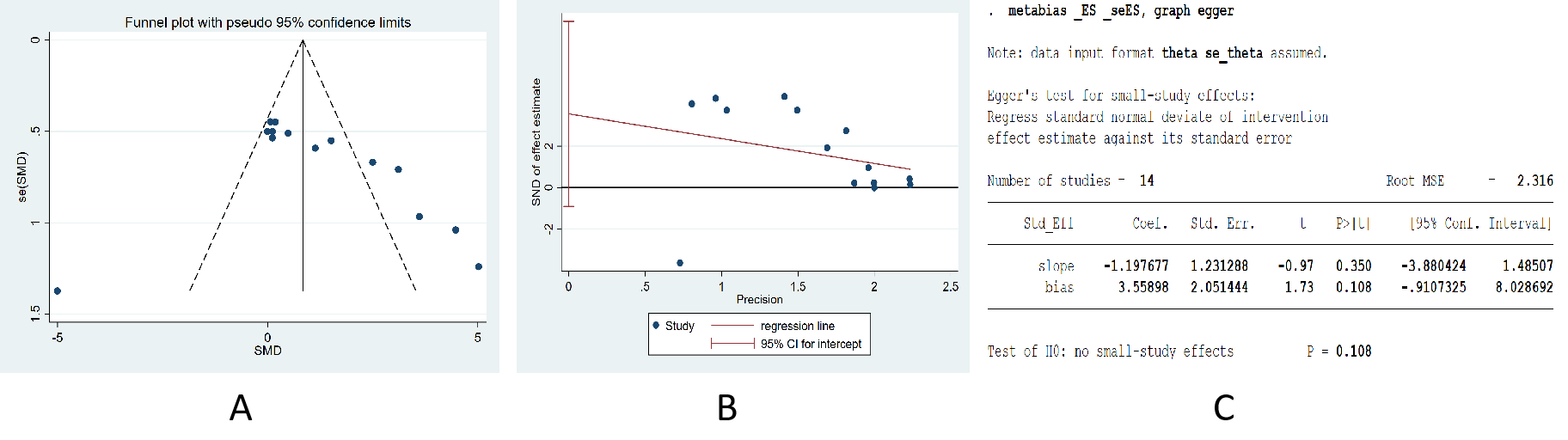


**Figure 9.** Publication bias of the effect of PDB extracts on **body weight**. (A) Funnel plot of publication bias; (B) Graph of egger^,^s test; (C) Result of egger^,^s test
